# Insights from the Convolution of Emission Inventories with Flux Footprints from Tall Eddy Covariance Towers at Three European Cities

**DOI:** 10.1021/acsestair.6c00148

**Published:** 2026-06-02

**Authors:** Betty Molinier, Patrick Aigner, Dominik Brunner, Jia Chen, Andreas Christen, Lionel Constantin, Hugo Denier van der Gon, Rainer Hilland, Daniel Kühbacher, Junwei Li, Robert Maiwald, Ingrid Super, Sanam N. Vardag, Natascha Kljun

**Affiliations:** † Centre for Environmental and Climate Science, 5193 Lund University, 223 62 Lund, Sweden; ‡ Professorship of Environmental Sensing and Modeling, 9184 Technical University of Munich, 80333 Munich, Germany; § Empa, Laboratory for Air Pollution/Environmental Technology, 8600 Dubendorf, Switzerland; ∥ Environmental Meteorology, University of Freiburg, 79085 Freiburg, Germany; ⊥ Netherlands Organisation for Applied Scientific Research, 3584 Utrecht, Netherlands; # Institute for Environmental Physics, 9144 Heidelberg University, 69120 Heidelberg, Germany; ∇ Heidelberg Center for the Environment, Heidelberg University, 69120 Heidelberg, Germany

**Keywords:** urban, greenhouse gases, inventory development, footprint model, temporal profiles, source sectors

## Abstract

Climate change is primarily driven by anthropogenic emissions of greenhouse gases. Reducing these emissions globally requires a massive effort at the individual, city, and national scales. Urban areas are hotspots of anthropogenic emissions, given the high density of human-based activities, and clarity on the variations of emissions in these areas will enable effective targeted reduction plans. However, efforts to do so are hampered by a lack of direct measurements of temporal and spatial trends of the emissions. This study combines emission inventories with flux observations and footprint modeling in three pilot sites for urban emission studies (Zurich, Munich, and Paris) with a focus on carbon dioxide (CO_2_), carbon monoxide (CO), and methane (CH_4_). Results indicate that the sectors contributing most significantly to CO_2_ fluxes, stationary combustion and road transport, are consistent across the cities and require future reduction plans to target winter months and daytime hours (05:00–17:00 UTC). The sectors contributing to CO and CH_4_ fluxes vary by city and do not always have consistent seasonal or diurnal patterns. Results also provide a basis for improving emission inventories and temporal scaling factors across sites and species in order to achieve better agreement with observations.

## Graphical abstract

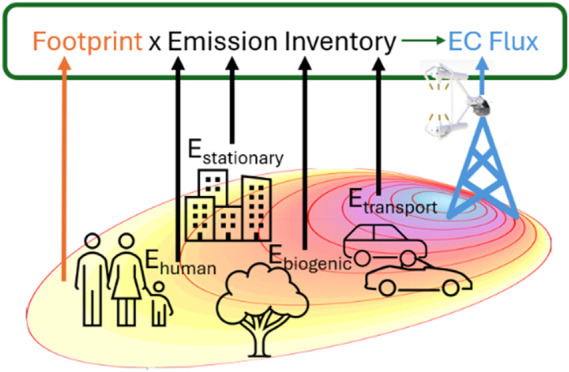

## 1 Introduction

Climate change, driven by anthropogenic greenhouse gas (GHG) emissions, has effects that are felt to different extents around the world.^1–3^ It can result in extreme temperatures, sea level rise and flooding, more frequent and more devastating natural disasters, etc.,^4,5^ all of which have a direct impact on human health and adaptability.^6^ Current climate change policies are not enough to reduce anthropogenic emissions to a level that prevents more global warming.^7–9^ Humans in general need to change their behaviors in order to address the issue of rising GHG emissions as well as mitigate their effects.^1,7^ While this must be a collective effort, it also must be undertaken at individual, city, and national levels to have an effect. Examining the sources of these emissions on a localized scale is crucial to understanding how to stop their increase and mitigate the imminent effects of climate change on a global scale because cities often report different repercussions and affected sectors.^10^

Emission inventories have been implemented throughout the world to enable sectorial reporting of different GHGs and air pollutants emissions.^11–13^ While there are standards in the European Union for national emissions reporting,^14^ different entities may use different methods depending on availability, computations, and/or interpretations of the standards,^15,16^ which can complicate the comparability of the inventories themselves. This becomes increasingly apparent at the city level^17,18^ as reporting frameworks continue to be proposed and altered.^19^ For example, private industries and public entities have to report their emissions, but individuals do not, leading to the need for proxy data. Other times, third parties may develop city-level inventories, but may not have access to all the information they need.^20^ Additionally, sources of anthropogenic GHGs are diverse and can be difficult to quantify^21^ and as a result some GHGs, such as methane (CH_4_), are often underreported in emission inventories.^22–25^

Although limitations exist, it is possible to use measurement techniques and models to assess city-level emission inventories and develop viable plans to move forward with climate change mitigation. The eddy covariance (EC) flux measurement approach has been employed in many sites, with well-established networks extending across Europe as well as Asia and North America.^26–29^ While primarily used in areas with homogeneous land cover, such as forests or cropland,^26^ several flux towers have been established in urban areas as well.^30–33^ The turbulence parameters measured at any of these past or existing towers can be used as inputs in flux footprint models, to identify the spatially varying source area, or ‘footprint’, of a flux signal at a given time step.^34^ Similar to the combination of the flux footprint with vegetated land cover types for interpretation of the observed fluxes,^35,36^ the flux footprint can be combined with emission inventory data to yield a modeled flux value.^37^ Previous work has implemented this combination for CO_2_ measurements at one tower and emissions estimates from one inventory to evaluate observations,^38^ fine-scale emission inventories,^39^ effects of COVID,^40^ and source decomposition^41^ with different footprint models with success. Helfter et al. expanded the methodology to CO and CH_4_ to evaluate how well emissions were characterized in London.^42^

This paper presents a case study for this methodology using observations from three urban EC flux towers in Zurich (Switzerland), Munich (Germany), and Paris (France) of the Pilot Applications in Urban Landscapes (PAUL) project, a.k.a ICOS Cities^31^ in combination with the Flux Footprint Parameterisation (FFP) model.^34^ It evaluates both bottom-up and downscaled emission inventories and shows how the methodology can be implemented to provide concrete steps toward both improvement of city-level emission inventories and development of unique, local emission reduction plansplans.

## 2 Materials and Methods

### 2.1 Site Description and Instrumentation

The tall urban EC flux towers of the ICOS Cities project are located in the following cities: Zurich, Switzerland (“Hardau”, HAR); Munich, Germany (“Oberpostdirektion”, OPD); and Paris, France (“Romainville”, RMV). Site characteristics, including tower location and measurement heights, are summarized in Lan et al.^43^ Briefly, an integrated CO_2_ and water vapor (H_2_O) open-path gas analyzer and 3D sonic anemometer (IRGASON, Campbell Scientific, Inc., Logan, UT, USA)^44^ was installed at each tower to measure CO_2_ and H_2_O fluxes continuously from their respective installation date to the end of the project. Details on data processing and quality control can be found in Lan et al. and Vitale et al.^43,45^ Additionally, a high-frequency closed-path multispecies gas analyzer (MGA-7, MIRO Analytical, Wallisellen, Switzerland)^46^ was rotated between sites throughout the campaign for roughly six months (Table 1) to measure fluxes of trace gases, including CO and CH_4_. More details on data collection and processing can be found in Hilland et al.^47^ The site-specific Ecosystem Thematic Centre (ETC) Level 2 flux data sets for the years 2022–2024 ^48–50^ at 30 min resolution for all sites were used in this study for turbulence and CO_2_ flux measurements, and the ICOS Fully Quality Controlled Observational Data (also Level 2) products were used for the fluxes of coemitted species (CO and CH_4_).^51–53^ Flux footprints using the Level 2 ETC data are publicly available^54^, and example visualizations can be found in Figures S1–S3. The relevant area extent used for each tower is 12 km × 12 km for HAR, 15 km × 15 km for OPD, and 16 km × 16 km for RMV. More details can be found in Molinier & Kljun (2024).^55^

**Table 1:** Flux Sampling Duration for the Three PAUL Urban Flux Towers

city	ID	inlet height (*z*_m_, m agl)	IRGASON sampling duration	MGA-7 sampling duration
Zurich	HAR	111.8	Jul 2022–Dec 2024	Aug 2022–Mar 2023
Munich	OPD	85.0	Jan 2023–Dec 2024	Jul–Dec 2024
Paris	RMV	102.5	Feb 2023–Dec 2024	Jan–Jun 2024

### 2.2 Flux Footprint Parameterisation

The Flux Footprint Parameterisation (FFP)^34^ was implemented for each site to establish the area contributing to the measured gas flux at any given time within the selected area extent over the course of each site’s selected measurement duration for the IRGASON (Table 1). The MGA-7 sampling duration at each site occurred within the same measurement period, and the data were processed such that the time stamps were the same as those of the IRGASON data sets, so the resulting footprints are applicable to CO_2_ fluxes as well as to coemitted species fluxes. The FFP enables rapid computation over a long time period (>one year) with minimal limitations imposed by physical conditions or scenario validity.^34^ It is hence one of the few flux footprint models valid for tall towers, and despite not having been adapted to urban terrain, it accounts for roughness length explicitly if urban roughness length is properly considered. The heights of the measurement inlets surpass the threshold of 2*h* (where *h* is the height of the surface canopy) at all sites,^56–58^ indicating that they should also be well above the roughness layer^59^ which should allow for reasonable predictions from FFP despite not meeting the assumption of homogeneous flow. The resulting footprint represents the probability of any tracer released at a specific grid cell within the area of interest passing through a given sensor location. In practice, the footprint function value attributes each grid cell with a weight or relative contribution to the total flux. The model can be run at the temporal resolution of its input data and over relevant time scales for an aggregated footprint. For the purposes of this study, the model was run at a time resolution of 30 min, which is the same resolution as the processed flux data from both the IRGASON and MGA-7, and at a spatial resolution of 10 m.

The FFP requires input data measured at or derived for the tower location, which are explained in detail in Kljun et al.^34^ Briefly, inputs measured at the tower location are wind direction (*w*_d_, °), the standard deviation of lateral velocity fluctuations (σ_v_, m s^–1^), friction velocity (u_*_, m s^–1^), and the Obukhov length (*L*, m). The boundary layer height at each tower location was obtained from the ECMWF ERA5 reanalysis data,^60^ and the roughness length and displacement height maps within the relevant footprint extent were derived from building and vegetation height maps at each site in addition to the meteorological parameter inputs measured at the towers at each time step (cf. SI). Details regarding footprint quality assurance and flagging can be found in Molinier & Kljun.^55^ While many footprint models exist^61^ and their performance relative to each other can vary,^62–65^ the FFP was chosen for this study because: (1) its applicability over a wide range of stability regimes and measurement heights is needed for use with tall EC towers; (2) it can be run with input values that are measured at EC towers; (3) it directly considers dynamic estimates of roughness length and displacement height; (4) it enables rapid footprint computation (∼days) for long time series of EC flux data (∼years); and (5) it shows better performance regarding peak flux estimation than other models.^34^

### 2.3 Emission Inventories

An annual emission inventory for the year 2022 cataloguing sector-based emissions of CO_2_, CO, and CH_4_ was developed at 100-m resolution for every city with each grid cell containing sector-based annual emissions of each species of interest. This study employed the latest emission inventories available from the entities described in the following paragraph. The annual emission value of each species was extracted by emission sector. Twelve different sectors were included, following the Gridded Nomenclature For Reporting (GNFR) sector definition (Table S1). All three inventories also include CO_2_ from human respiration as a 13th category (Sector O in Tables S3–S5).

In contrast to the highly standardized flux measurement, each inventory was developed with a different approach, offering an opportunity for comparison. The city of Zurich provided its own inventory, while for Paris, an inventory developed entirely by a noncity affiliated project partner (Netherlands Organisation for Applied Scientific Research, or “TNO”) was used in this study. A new inventory was compiled for the city of Munich as part of the PAUL project. The cities themselves have access to more data than TNO, making this a useful case study for evaluating what resources are actually required for accurate emissions estimates and how to optimize them. Since (1) the observations at each tower were measured by the same instrument type and underwent the same quality control process, and (2) the approach to the footprint modeling and convolution with the inventories is the same, the comparison of the model results with the observations should enable a comprehensive assessment of the inventory development methodologies over the study area of each site.

#### 2.3.1 Zurich

The emission inventory for 2022 used in this study for Zurich was developed by combining data from MapLuft (city inventory developed by the city of Zurich) and the Swiss national emission inventory,^66,67^ via the emiproc package^68^ in Python at 100-m resolution. Emiproc enabled regridding and regrouping of sectors to the GNFR sectors. In addition to the 12 GNFR emission sectors listed in Table S1 and human respiration (Sector O), this inventory also contained an ‘Other’ sector (Sector R in Tables S3–S5). Emissions from human respiration were estimated with emiproc based on a building occupancy data set and using emission factors from literature.^68,69^ Sector R accounted for emissions from smoking, fireworks, zoo and household animals, accidental fires, thunderstorms, and some other natural emissions.

#### 2.3.2 Munich

Munich’s emission inventory for 2022 ^70^ was compiled using a hybrid approach at 100-m resolution. For all main emitting sectors (public power, stationary combustion, road transport, and human respiration), local data was collected from the city, and bottom-up methodologies were applied. Industrial process emissions were considered negligible within city borders, which is why emissions from sector B (industry) are not explicitly discussed. For some industrial facilities, self-reported gas and oil consumption values were included, and heating emissions for buildings in industrial areas were generally covered in sector C. The remaining sectors were taken from the European downscaled inventory to obtain a complete inventory, as detailed in the following section for Paris. This is also applicable for the methane emissions included in the inventory, which were computed by TNO.

#### 2.3.3 Paris

For Paris, the local bottom-up emission inventory was only available at 500-m resolution. To maintain consistency of spatial resolution among city inventories, the European emission inventory CAMS-REG v8.1 HR for 2022 was downscaled to a 100-m resolution for the study area. The CAMS-REG HR emission inventory was developed following a similar approach as the CAMS-REG emission inventory^71^ but at a higher resolution (1/60° longitude x 1/120° latitude). For the downscaling, open-access spatial data sets were downloaded (Table S1) and for most emission sectors multiple data sets were combined or scaled to create spatial proxy maps that are representative of the emission activities for each sector separately. The total emissions were redistributed using proxy maps at high resolution. The downscaling was done for all CAMS-REG grid cells that (partly) fall within the city domain to ensure the conservation of emissions. Note that point sources in the CAMS-REG emission inventory have exact coordinates and were therefore not downscaled; more details can be found in Super et al.^72^

#### 2.3.4 Temporal Scaling

The above emission inventories have been reported on an annual basis. For the combination with the footprints, it is necessary to develop hourly fluxes using temporal profiles or ‘scaling factors’ for the (1) month of the year, (2) day of the week, and (3) hour of the day. Each of these three scaling factor profiles was developed for the 12 GNFR emission sectors as well as the human respiration and ‘other’ sectors, and can be applied to any gas of interest. The three sets of temporal profiles were then multiplied to obtain a sector-based temporal scaling factor for every time step over the study period. The methodology for developing the temporal profiles for each city is explained below.

##### 2.3.4.1 Zurich

The temporal profile for stationary combustion was derived from heating degree days using temperature data from a site in the center of Zurich (Kaserne) and domestic hot water demand profiles from the Swiss Society of Engineers and Architects (SIA) norms. Traffic profiles came from hourly traffic data for the Hardau area provided by the city of Zurich. Human respiration profiles came from SIA norms of building occupancy, for both residential and office buildings. Other profiles (industrial, fugitives) were built based on custom cyclic profiles from TNO. These profiles are given only for the whole city, so there is no spatial variation in the temporal distribution.

##### 2.3.4.2 Munich

Instead of using default temporal factors for public power, stationary combustion, and road transport, profiles specific to activity data in Munich were derived.^73,74^ The heating degree day function for Munich was derived using hourly district heating load data provided by the local utility company. Since district heating represents close to 40% of total heating in Munich, and heating behavior is based on general human activity rather than the locally present heating type, these patterns were assumed to represent overall residential heating behavior. The resulting heating degree day function considers outside temperature, season, weekday/weekend, and time of day to predict hourly heating activity that is representative of local habits. Temporal profiles for the road transport sector were calculated using DRIVE,^74^ which combines traffic counting data from multiple counting stations within the city area and provides species-specific temporal profiles. They depend on the actual ambient temperature and account for the nonlinear relationship between traffic volume and emission factors, as a high traffic volume typically leads to congestion, resulting in proportionally higher emissions.

For the purposes of this study, road transport emissions for all species of interest were divided into ‘hot’ emissions and ‘cold start’ emissions, which have their own temporal profiles specific to temperature and species. This differentiation is relevant to CO emissions only. Additionally, three profiles for different scenarios regarding human respiration in Munich were calculated: (1) indoors (residential), (2) indoors (nonresidential), and (3) outdoors. Only the total of each sector is presented for the purposes of this study. Default temporal scaling factors^75^ were used for the remaining sectors.

##### 2.3.4.3 Paris

Default temporal scaling factors^75^ for month of year, day of week, and hour of day were used for all sectors. Generally, each temporal profile is constructed to reflect patterns observed in long-term data sets for different aggregated emission sectors.^75^ However, these factors are not country- or region-specific, do not include drivers such as temperature, and do not capture differences between emission patterns in urban areas vs rural areas.^75^ Because human respiration is not a GNFR sector, no default profile has been developed for this emission category. As this sector is not split by location (for example, work vs home), it was assumed that there are no diurnal changes in human respiration emissions. Emissions were also assumed to be constant throughout the year due to continued tourism and lack of information on travel patterns in Paris. Despite these limitations, the generalizability of these default temporal profiles allows for their application across multiple species, including the three examined in this study.

### 2.4 VPRM Model

The Vegetation Photosynthesis and Respiration Model (VPRM) is a satellite-based assimilation scheme to estimate net ecosystem exchange of CO_2_.^76^ Briefly, this model builds on the Vegetation Photosynthesis Model,^77^ which uses satellite-based indices such as the Enhanced Vegetation Index (EVI) and the Land Surface Water Index (LSWI) to model gross ecosystem exchange (GEE). Adding a respiration (R) component, modeled as a function of air temperature, subsequently enables derivation of net ecosystem exchange (NEE),^76^ which is defined as NEE = −GEE + R. According to this definition, positive NEE indicates emission while negative NEE suggests uptake via photosynthesis, which can only occur when there is daylight. The VPRM was set to take eight vegetation classes derived from several land cover databases^78–85^ into account (evergreen forests, deciduous forests, mixed forests, shrublands, savannas, croplands, grasslands, and others), requires input data regarding atmospheric conditions,^86^ and is adapted to use satellite data from Sentinel-2.^87–89^ While adaptations have been made to the model for the urban context,^90–92^ the original version was used for the purposes of this analysis as the urban and modified outputs did not differ significantly from the original.^93^

### 2.5 Convolution and Modeled Fluxes

The spatial extent and resolution of the emission inventories were adapted using QGIS Desktop v.3.42.0 (‘Münster’; *QGIS Association*), by rasterizing the vector layers of each emission sector for each species and subsequently regridding those raster layers in MATLAB 2024b (*The Mathworks, Inc.*) to 10-m resolution over the relevant footprint extent for each city using nearest-neighbor interpolation. All maps for emissions (kg a^–1^) from each sector and species of interest were convolved with the flux footprints (m^–2^) and temporal scaling factors in MATLAB as well. The resulting products of the convolution were converted to fluxes (μmol m^–2^ s^–1^) by using the molar mass (g mol^–1^) of each species and unit conversion factors.

Per definition, any site domain can only capture a fraction of the footprint. The cumulative footprint weight within the study areas was usually 80% or higher, so it was assumed that the remaining 20% (or less) of the area contributing to the flux signal contained similar emission sector contributions as the included area.^39^ Therefore, the summed footprint values were normalized to unity. Total modeled anthropogenic fluxes of each species were calculated by summing fluxes across all sectors. All data were converted to UTC prior to convolution to ensure a standard time across sites and data products.

## 3 Results and Discussion

### 3.1 Model Comparison with Observations across Cities

To elucidate how well the convolution of the footprints and emission inventories outlined in Section 2 agrees with the flux observations at each site, Figure 1 (CO_2_) and 2 (CO and CH_4_) compare the mean monthly diurnal total modeled fluxes within the footprint with mean monthly diurnal observed fluxes for each month of the relevant measurement campaign as described in Table 1. In addition, Figures S4–S12 present scatter plots and Spearman correlation values by wind direction at 30 min resolution, and Figures S13–S15 present the hourly mean modeled fluxes for every GNFR sector and the hourly mean observed fluxes for each month to identify over- or underestimation of emission sectors as compared to the observations. Table S2 also presents correlations at 30 min resolution under different temporal, temperature, and wind direction circumstances.

**Figure 1:**
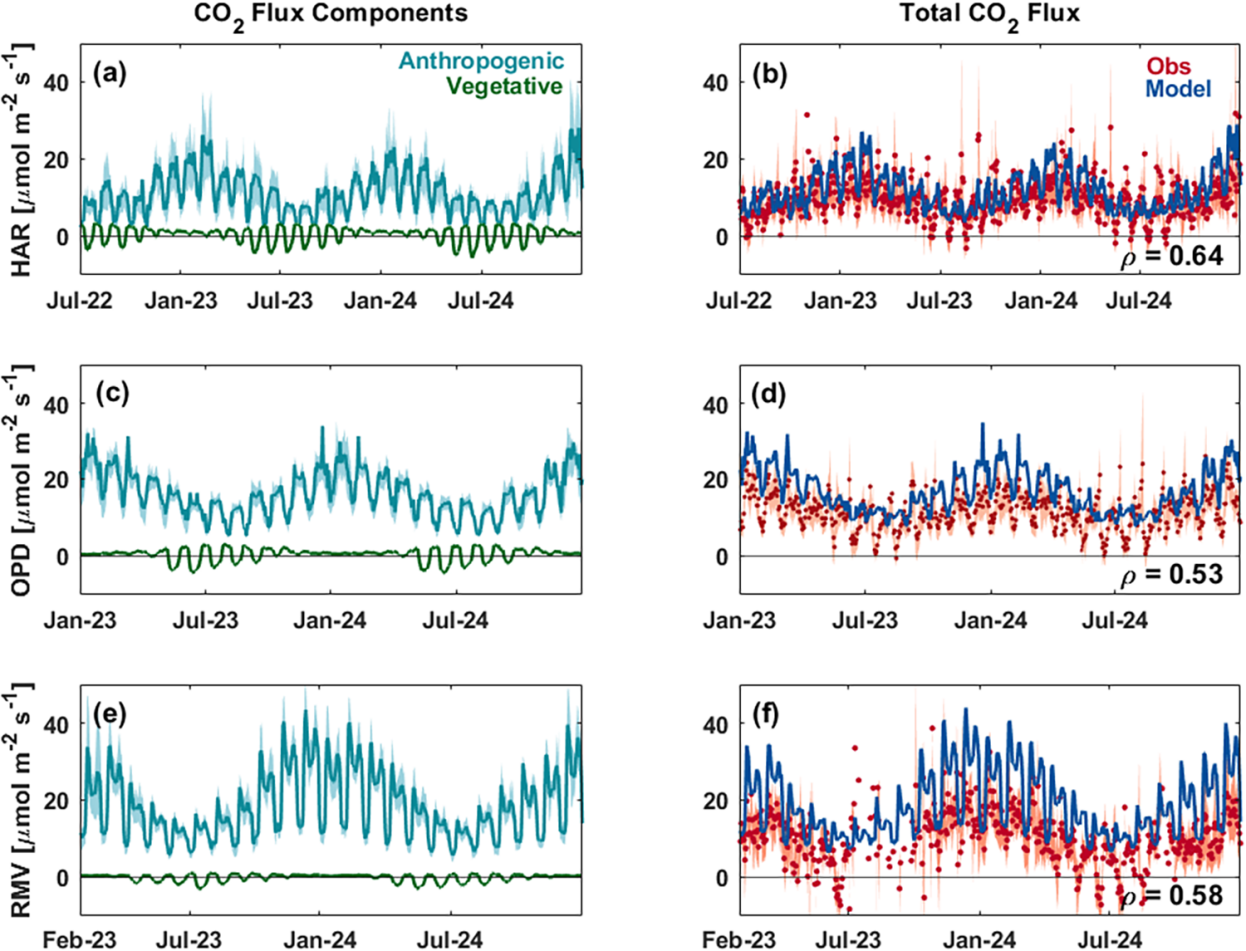
Comparison of mean monthly diurnal footprint-modeled anthropogenic CO_2_ fluxes (teal line), mean modeled biogenic CO_2_ fluxes (green line), and total modeled CO_2_ fluxes (blue line) to CO_2_ flux observations (red points) from ETC L2 Flux data in UTC at (a, b) HAR (Jul 2022–Dec 2024), (c, d) OPD (Jan 2023–Dec 2024), and (e, f) RMV (Feb 2023–Dec 2024). All shaded regions indicate interquartile range; while the interquartile range for VPRM was calculated and plotted, they were too low to be visible.

Figure 1a,c,e show the modeled monthly diurnal anthropogenic and biogenic CO_2_ fluxes at HAR, OPD, and RMV, respectively. Anthropogenic fluxes are higher in winter than in summer at all sites and show a strong diurnal pattern, with fluxes typically being higher during daytime hours than at night; this is expected given higher human activity during the day. The comparison of individual emission sector contributions to the total flux signal for the full measurement duration (Figure S13) shows that at all three sites, stationary combustion is the most significant contributor to the magnitude and temporal behavior of the modeled anthropogenic fluxes. From the VPRM results, the dependence of the biogenic fluxes on season and daylight is evident at all three sites, and as expected, the signal in winter months is nearly (but not equal to) zero.

Figure 1b,d,f compare the summation of the modeled anthropogenic and biogenic CO_2_ fluxes and the mean monthly diurnal observed fluxes. Observations tend to fall between 0 and 30 μmol m^–2^ s^–1^, with some exceptions in summer 2023 at RMV. The Spearman correlation coefficient (ρ) of the model and observations falls within the range of ‘moderate’ agreement, which is 0.38 ≤ ρ < 0.68. The model agrees best with observations at HAR (ρ = 0.64), with some seasonal overestimation in winter (Dec–Feb) by up to a factor of 3; at OPD and RMV, the seasonal pattern in the observations is well-captured by the model, but the magnitude is systematically overestimated by 2–4×. The story is slightly different at RMV (Figure 1f)—the observations in summer 2023 are more scattered than at the other sites, possibly due to the fact that not all data collected during rainfall were flagged correctly and removed in the quality control process. The figure indicates that the combination of the footprint convolution with the emission inventory and the VPRM results does not fully explain the discrepancy with observations. It should be noted that the observations themselves may also be an important factor in the discrepancy because of the impacts of choices made in instrumentation, processing software, and spectral correction schemes.^43^ However, because the same methodology and instrumentation were used across sites, any resulting uncertainty is not considered further in this paper. When comparing the fluxes of each individual emission sector to the observations (Figure S13) rather than the total, the sector(s) that may cause the discrepancy between the model and the observations can be identified: stationary combustion is the most significant contributor to the flux signal and is equivalent to or higher than the observations, which indicates that this sector may be an important driver in the aforementioned model discrepancy.

The measurement campaigns for the coemitted species were roughly six months each (Table 1), hence their comparison occurs over a shorter time period than for CO_2_. These measurements are not simultaneous across sites as the MGA-7 was rotated among them. Figure 2a,c,e display CO fluxes while Figure 2b,d,f display CH_4_ fluxes. Observed CO fluxes tend to fall between 0 and 0.10 μmol m^–2^ s^–1^, with a few exceptions in Jan 2024 at RMV, while observed CH_4_ fluxes tend to fall only between 0 and 0.05 μmol m^–2^ s^–1^ (though there are a few exceptions at all three sites). The modeled CO fluxes at all sites show ‘strong’ agreement, which consists of ρ ≥ 0.68. The correlation between the CH_4_ modeled and observed fluxes is ‘weak’, or below 0.38, at all sites; at RMV, the correlation is weakly negative. The mean and interquartile range of the modeled anthropogenic fluxes for each species indicate that the model underestimates both species as compared to the observations at HAR by 2–3× (CO) and by up to a factor of 15 (CH_4_) (Figure 2a,b). This underestimation is partially because emissions from a water treatment plant and from leaks in the gas system were not considered in the inventory. At OPD, the agreement between the modeled and observed CO fluxes is similar to that at HAR, but here the modeled values are consistently higher than the observed values; CH_4_ fluxes tend to be underestimated by the model by up to 6× with some exceptions (Figure 2c,d). Lastly, at RMV, CO fluxes tend to be overpredicted by the model (Figure 2e) while CH_4_ fluxes are overpredicted only in some months, sometimes by more than a factor of 4 (Figure 2f). This would suggest that temporal profiles specific to these species may need to be developed. The pattern for CO flux observations appears to be captured by the model, but the magnitude and range are not fully encapsulated and can be overestimated by up to a factor of 10.

**Figure 2:**
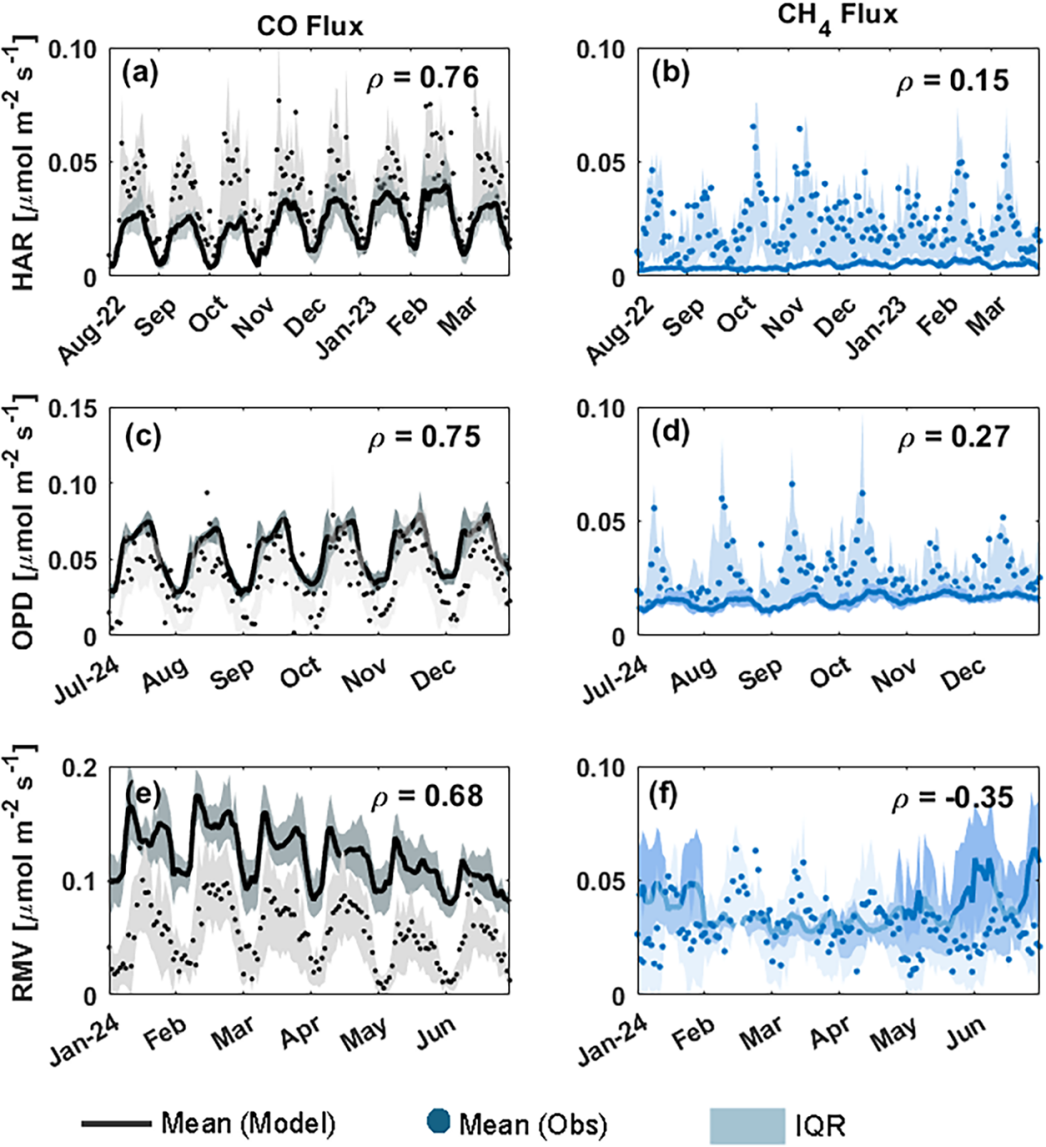
Comparison of the mean and interquartile range (IQR; shading—dark for model, light for observations) of modeled (line) anthropogenic fluxes of CO (black) and CH_4_ (blue) with MGA-7 observations (points) in UTC at (a, b) HAR (Aug 2022–Mar 2023), (c, d) OPD (Jul–Dec 2024), and (e, f) RMV (Jan–Jun 2024). Units for the *y*-axis are the same across all three panels; please note the *y*-axis range for CO differs across sites.

The modeled CO signal is dominated by stationary combustion and road transport at HAR, by on- and off road transport at OPD, and by off road transport followed by a combination of stationary combustion and road transport at RMV (Figure S14). CO is the product of incomplete combustion which is highly fuel and technology dependent. At HAR, the CO flux observations demonstrate a strong diurnal but no seasonal pattern, indicating that the main contributors to the signal should have a strong diurnal pattern, such as road transport emissions. The model captures this pattern, but not the amplitude of the observations. Emission sectors with a strong seasonal pattern may not be as significant at HAR in Zurich. The CO flux observations at OPD exhibit similar behavior as at HAR. At RMV, the observations show a strong seasonal pattern that is similar to the observed pattern in stationary combustion. While the agreement between the model and observations for CO at RMV meets the criteria for ‘strong’ agreement, it still shows the lowest performance out of the three sites, indicating that temporal profiles specific to Paris, and potentially specific to CO, may need to be developed for better agreement.

Figure S15 shows that CH_4_ is dominated by stationary combustion at HAR in winter months; however, the same species is dominated by waste and fugitive emissions at the remaining sites instead. Figure S15a also indicates that the observations are so much higher than the model results that it is difficult to discern which emission sector may require further investigation. As waste and fugitives were the main contributors in the other cities, it may be worthwhile to start with these sectors at HAR to determine whether this difference arises from activities within the footprints or from how the emission inventory accounts for methane. The diurnal pattern in CH_4_ flux observations at OPD is not as clear as at the other sites, but as the waste sector does appear to have an observable diurnal trend despite a stable temporal profile, particularly in summer months; this is likely due to the footprint extent and direction. The trace for waste emissions at RMV appears to agree with the magnitude of CH_4_ observed fluxes, with the notable exception of Feb–Mar 2024, indicating that it may truly be the main contributor to the signal and that the contribution from fugitive emissions may be overestimated in the inventory. It is worth noting that accounting for methane emissions in inventories is generally quite difficult and the methodology to do so may need to be discussed further.

### 3.2 Temporal Trends in Modeled Anthropogenic Fluxes across Cities by Sector

As emission inventories are often provided on an annual basis, temporal scaling factors are needed to provide information on how emissions vary throughout the year (cf. Section 2.3.4). The comparison of the model to the observations in the previous section has shown how well this methodology works for each species and has highlighted areas for improvement. As a next step, Section 3.2.1 discusses seasonal patterns while Section 3.2.2 examines diurnal patterns at each site in order to demonstrate how this methodology may be a useful starting point for identifying emission sectors to target in localized reduction plans once emission inventories and temporal scaling factors are evaluated.

#### 3.2.1 Seasonal Trends

To compare seasonal trends in footprint-weighted modeled anthropogenic fluxes across cities, a one-year period was chosen during which turbulence measurements were collected concurrently at all three sites (Mar 2023–Feb 2024). Means and standard deviations by emission sector for the selected measurement period can be found in the SI. Figure 3 displays a comparison of the monthly average estimated anthropogenic fluxes for (a) CO_2_, (b) CO, and (c) CH_4_ at all three sites (HAR, OPD, and RMV) by GNFR emission sector. The contributions from the shipping, aviation, and agri-other sectors were negligible at all sites and therefore not included. Flux estimates for CO_2_ at RMV tend to be highest across cities with the exception of the Spring 2023 months (Figure 3a), up to a factor of 2 higher than at HAR and anywhere from 5 to 20% higher than the CO_2_ flux estimates at OPD. Flux estimations for both CO and CH_4_ are also consistently highest at RMV by a factor of 2 up to a factor of 5, followed by OPD and then HAR. Estimated CO fluxes at HAR show a seasonal dependence similar to the observed CO_2_ trend in Figure 3a, but it is not as strong. Estimated CO fluxes at OPD do not show a seasonal pattern; at RMV, fluxes do show a seasonal trend originating from industrial and stationary combustion.

**Figure 3:**
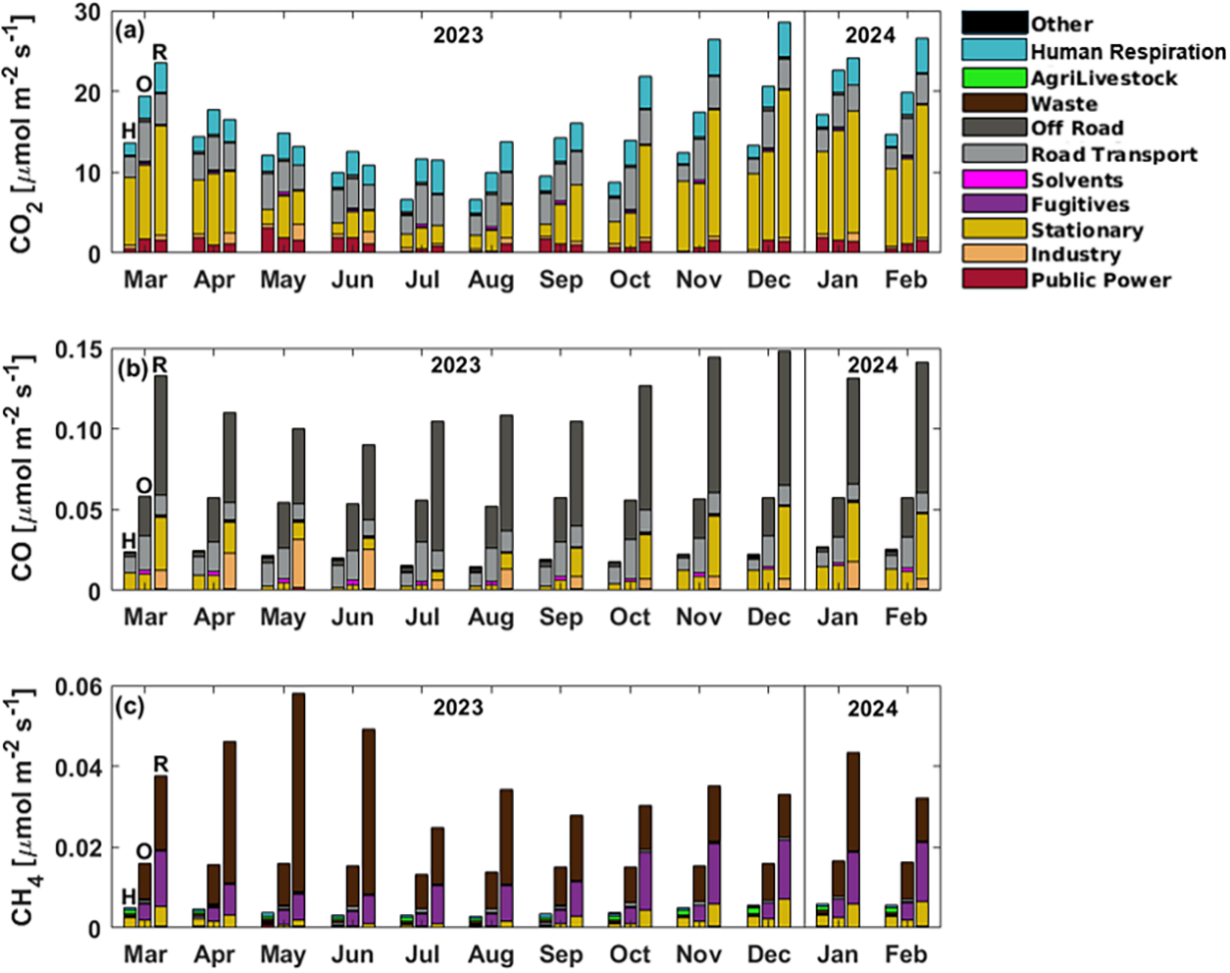
Modeled average anthropogenic footprint-weighted flux (μmol m^–2^ s^–1^) estimations for (a) CO_2_, (b) CO, and (c) CH_4_ across all cities from Mar 2023–Feb 2024 by emission sector (color-coded, see legend). For each month, the first bar represents results at HAR (H), the second bar OPD (O), and the third bar RMV (R).

The dominant CO_2_ (Figure 3a) emission sector at all three measurement sites is stationary combustion (yellow bar) followed by road transport (light gray bar) and human respiration (blue bar). The contribution from stationary combustion displays a seasonal pattern, especially at HAR, with a high contribution in the winter months (Dec–Feb) and a lower contribution in the summer months (Jun–Aug). This pattern is expected as people are more likely to heat their homes or workplaces in colder months. Contributions from road transport may fluctuate a little (see SI for means and standard deviations), but are mostly consistent from one month to the next. This is also expected as traffic patterns in cities tend to depend more on the hour of the day and the day of the week than on the month of the year.^94,95^ Typically, fluxes from stationary combustion are 3–4× higher than from road transport at all three sites in winter, but are generally equal to or only slightly higher than fluxes from road transport in summer. Human respiration fluxes also do not have a seasonal dependence. Contributions from other sectors are quite low and can be examined further in Table S3.

The dominant CO (Figure 3b) emission sectors at HAR are road transport followed by stationary combustion and off road. This differs slightly at OPD and RMV, where the dominant sectors are road transport plus off road and off road plus stationary combustion, respectively. The stationary combustion and road transport contributions to CO fluxes follow a similar seasonal pattern (or lack thereof) as the contributions to CO_2_ fluxes. Contributions from off road transport to the CO signal at OPD remain relatively steady but are generally higher at RMV by a factor of 3 or more than at OPD. The flux signal at RMV appears to receive a higher contribution from industrial sources compared to HAR. Further information regarding contributions from other sectors can be found in Table S4.

The dominant CH_4_ (Figure 3c) emission sector at HAR is stationary combustion (which again displays seasonal dependence), followed by human respiration and agri-livestock, which both appear consistent throughout the year. As some sources are missing from the inventory, it is difficult to discuss this further. This differs from the pattern observed at both OPD and RMV, which indicates that the dominant CH_4_ emission sector at both sites is waste followed by fugitive emissions. It should also be noted that neither account for CH_4_ from the ‘human respiration’ sector. Waste emission contributions to the flux signal at RMV are quite high in the spring months and are often 3–4× higher than waste contributions at OPD. They tend to be higher in winter months than in summer months at RMV while the contributions at OPD tend to be consistent throughout the year. Because waste emissions from methane are distributed using population data,^71^ it is likely that these patterns are dependent on whether or not the footprint extent and direction correspond to a densely populated area. Fugitive emissions do not show any seasonal pattern, as expected from the temporal profiles, but seem to be lowest in spring at RMV.

#### 3.2.2 Diurnal Trends

In the following, diurnal trends in the most relevant emission sectors are explored for CO_2_, CO, and CH_4_ at all three study sites over two months of the year 2023 (Figures 4 and S16–S18). The hourly mean fluxes for a summer month (July) and a winter month (December) were chosen for examination in order to determine whether or not targeted reduction plans need to account for both diurnal and seasonal patterns for certain emission sectors. Figure S19 examines seasonal and diurnal patterns for the ‘Other’ sector at HAR.

**Figure 4:**
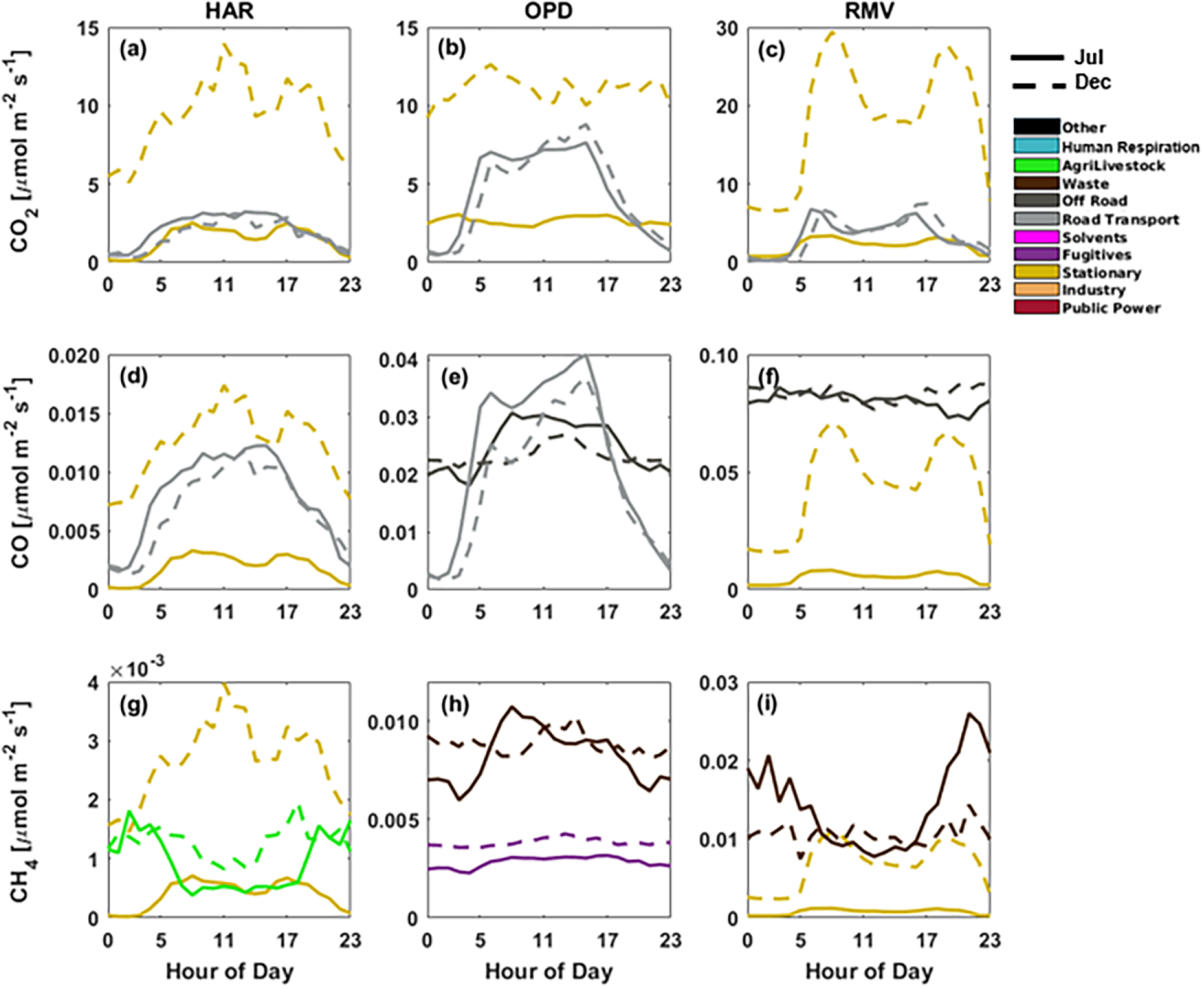
Modeled diurnal trends in Jul 2023 (solid) and Dec 2023 (dashed) for the two most dominant contributors to footprint-weighted (a)–(c) CO_2_ fluxes, (d)–(f) CO fluxes, and (g)–(i) CH_4_ fluxes across cities. Hours of Day are in UTC. To ensure visibility of trends, the *y*-axes show different ranges.

Figure 4a–c depicts stationary combustion (yellow) and road transport (light gray) for CO_2_ across all cities as these were shown above to be the highest two contributors to the flux signal at each tower throughout a calendar year. Patterns in stationary combustion show peaks at different times of day across cities in both months. At HAR, the flux diurnal patterns are similar in both seasons with the main difference being the magnitude, which is usually 5–10× higher in December than in July and has been previously discussed. At OPD, the stationary combustion diurnal profiles for both months show similar patterns as well but with smaller spikes in the December profile. The December fluxes are roughly 4× larger than the July fluxes. At RMV, the July diurnal profile appears relatively steady from 05:00–17:00 UTC due to the scale of the figure, but follow a similar pattern to the December profile, in which both a midmorning and a nighttime peak can be observed. The winter fluxes are significantly higher than the summer fluxes. The diurnal profiles for road transport are nearly identical in magnitude and behavior between months at each site, with the start of the morning peaks occurring an hour earlier in summer than in winter due to daylight savings.

Figure 4d–f display CO fluxes of stationary combustion and road transport at HAR, road transport and off road at OPD, and stationary combustion and off road at RMV. Diurnal profiles for stationary combustion and road transport at HAR mirror those seen in Figure 4a for CO_2_, which is expected as the same temporal profiles are applied to both species. At OPD, the road transport profiles for both months appear to follow a similar pattern as the CO_2_ road transport fluxes in Figure 4b, with the exception that the July fluxes are slightly higher throughout the entire day. Off road CO fluxes peak at different times of day (07:00 UTC in summer and 12:00 UTC in winter). At RMV, stationary combustion follows the same pattern as CO_2_, similarly to HAR and for the same reason. Off road CO fluxes are nonzero and remain steady throughout the day at the same level in both seasons.

Methane fluxes from stationary combustion and agri-livestock at HAR, from fugitives and waste at OPD, and from waste and stationary combustion at RMV are shown in Figure 4g–i. Stationary combustion diurnal profiles for both months are consistent with Figure 4a,d. Fluxes from agri-livestock are low during the day and higher at night for both July and December, stemming from the footprint source area and direction, but the order of magnitude is quite low compared to other sectors. The diurnal profile for waste in July at both OPD and RMV also shows more fluctuation than in December, with summer fluxes being higher during the day at OPD than at night while the inverse is true at RMV. The fluxes from fugitives at OPD are nonzero but steady throughout the day in both seasons. Methane fluxes from stationary combustion at RMV are low but have peaks occurring in the winter profile at the same hours as in the CO_2_ and CO diurnal profiles due to the use of the temporal profile across all species.

### 3.3 Key Insights from Model Comparison and Temporal Trends

The dominant emission sectors and their trends for each species at each site are dependent on the composition of sources within each study area. One measurement tower is unlikely to be representative of an entire city regardless of its size given that it cannot cover the entire area (cf. Section 2.1), which is important to consider as the differences between sites are examined further. For example, these trends indicate that the hours at which the emissions of CO_2_ and coemitted species from stationary combustion peak differ, which could (1) be a result of which emission sources are located and/or what human activities occur near the measurement site as well as meteorological conditions such as wind direction, (2) be an indication of methodological differences in the inventories and temporal factors for each city, or (3) genuinely be caused by city-wide differences in dominant emitters if it is assumed that the composition of sources varies by city rather than by location within each city. Regarding the first option, emission sources near the tower location are more highly weighted within the footprint and contribute more to the measured flux signal, but are not necessarily representative of the entire site or city. Other studies using the EC technique to examine emissions have also highlighted that the location bias^97^ of a footprint or source area resulting from where a flux measurement tower is installed is important to consider when explaining emission trends at a study site.^39,97–99^ Data availability and reporting requirements for entities within each city can differ,^18,100,101^ which could contribute to differences in annual emissions calculations for any of the inventories, regardless of how they were produced. The last option requires a spatial analysis that is outside the scope of this study.

One of the benefits of this study is the diversity of resources available from each pilot city, but this also introduced some limitations that should be addressed as this work continues. Because the emission inventories for each city were developed by different entities, the methodology or assumptions made for determining emissions of each relevant sector could differ depending on the available information and the interpretation of the definition of each GNFR sector. The use of local data is less prone to systematic bias, as city-specific characteristics such as district heating networks (which replace stationary combustion emissions) or a detailed analysis of local industrial emissions can yield a drastically different result compared to national downscaled inventories on the city scale. The temporal scaling factors also differed across cities, with some developed specifically for the city’s conditions^74^ while others were developed for application across Europe.^75^ A limitation of these default factors is that the stationary combustion profile only accounts for residential heating patterns and does not include commercial heating patterns. Using an emission inventory and temporal scaling factors developed by the city^102–104^ in which a flux tower is located is usually preferred as it leads to better model performance, as observed at HAR (Figures 1b and 2a) for CO_2_ and CO compared to other sites. However, more work needs to be done to better characterize emissions across sites and species regardless of inventory development methodology, as the model performed best at RMV for CO (Figure 2e) despite not performing well for other species at that site. Additional limitations from the modeling perspective include: (1) that there is no footprint model that is adapted for urban conditions; (2) that other models than FFP for flux footprints or VPRM for biogenic CO_2_ fluxes were not considered, but could be implemented in the future for comparison;^61,91,105^ (3) that 2023/2024 emission inventories and temporal profiles were not available at all sites for comparison with the 2023/2024 observations; and (4) the uncertainties within the FFP,^34^ the emission inventories, and the observations.^43^ Adding some strategically placed EC towers could provide enough information to evaluate emission inventories for the entire city, rather than a subset, which would enable a discussion regarding the impact of national reporting standards or local management practices on emission inventory development.

This study demonstrated the applications of flux footprints for urban sites and the robustness of the methodology for footprint and emission inventory convolution. The modeled anthropogenic CO_2_ fluxes showed the same magnitude and seasonal pattern as the flux observations at all three sites, but performed better at HAR than at OPD or RMV, which may be a result of differences in inventory and/or temporal factors used. The biogenic CO_2_ results from VPRM show the importance of biogenic emissions and sinks in urban areas, but discrepancies between the combination of the biogenic and anthropogenic modeled fluxes and the observations indicate that more work is needed to improve model agreement. The performance of the model with respect to coemitted species observations (CO and CH_4_) varied by site and hence by emission inventory. Anthropogenic CO and CH_4_ are more difficult to accurately quantify in an emission inventory compared to CO_2_ and were, for example, only a byproduct of the CO_2_ inventory compilation in Munich. This study also examined seasonal and diurnal trends of the modeled anthropogenic fluxes to highlight areas of improvement, both for inventory development and for emissions reduction. The presented approach can be applied to any city with an EC flux tower and emission inventory, and is a promising first step in identifying targets for localized reduction plans and climate change mitigation once emission inventories have been appropriately evaluated.

## Supplementary Material



## Notes

Spin-up procedure for calculating dynamic roughness length and displacement height, example footprints for one time step at each site, TNO emission inventory data used to calculate emissions for each sector, correlation plots and tables at 30 min resolution, results of model decomposition, mean and standard deviation tables for each species by emission sector at each site, diurnal profiles, and details on ‘other’ emissions at Zurich-Hardau (PDF)

## ○

Department of Earth and Environmental Sciences, Lund University, Lund 223 62, Sweden
