# Supplementary material for: Insights from the Convolution of Emission Inventories with Flux Footprints from Tall Eddy Covariance Towers at Three European Cities
Source: ACS EST Air. 2026 Jun 2;3(6):1664–76. doi: 10.1021/acsestair.6c00148 (PMC13270518; doi:10.1021/acsestair.6c00148)
Supplement: Supplementary file 1 [file ea6c00148_si_001.pdf]

Supporting Information for

Insights from the Convolution of Emission Inventories with Flux Footprints from Tall Eddy Covariance Towers at Three European Cities

Betty Molinier[1,*,‡], Patrick Aigner[2], Dominik Brunner[3], Jia Chen[2], Andreas Christen[4], Lionel Constantin[3], Hugo Denier van der Gon[5], Rainer Hilland[4,5], Daniel Kühbacher[2], Junwei Li[2], Robert Maiwald[6], Ingrid Super[5], Sanam N. Vardag[6,7], Natascha Kljun[1,‡]

[1]*Centre for Environmental and Climate Science, Lund University, 223 62 Lund, Sweden*
[2]*Professorship of Environmental Sensing and Modeling, Technical University of Munich, 80333 Munich, Germany*
[3]*Empa, Laboratory for Air Pollution/Environmental Technology, 8600 Dubendorf, Switzerland*
[4]*Environmental Meteorology, University of Freiburg, 79085 Freiburg, Germany*
[5]*Netherlands Organisation for Applied Scientific Research, 3584 Utrecht, Netherlands*
[6]*Institute for Environmental Physics, Heidelberg University, 69120 Heidelberg, Germany*
[7]*Heidelberg Center for the Environment, Heidelberg University, 69120 Heidelberg, Germany*

[*]Email: betty.molinier@mgeo.lu.se

[*]*Corresponding author*

[‡]*Now at the Department of Earth and Environmental Sciences, Lund University, Lund, Sweden*

SI Contains:

34 pages

5 tables

19 figures

SI Table of Contents

1. (*page S4*) – Roughness Length and Displacement Height Derivation
2. (*page S5*) – Example Flux Footprint Visualization
    a. **Figure S1.** Example footprint at Zurich-HAR.
    b. **Figure S2.** Example footprint at Munich-OPD.
    c. **Figure S3.** Example footprint at Paris-RMV.
3. (*page S7*) – TNO Emission Inventory Development
    a. **Table S1.** List of GNFR sectors in the emission inventories
4. (*page S8*) - Correlations at 30-min Resolution
    a. **Figure S4.** Scatter plots of observed and modelled CO_2 fluxes at 30-minute resolution at ZH-HAR.
    b. **Figure S5.** Scatter plots of observed and modelled CO fluxes at 30-minute resolution at ZH-HAR.
    c. **Figure S6.** Scatter plots of observed and modelled CH_4 fluxes at 30-minute resolution at ZH-HAR.
    d. **Figure S7.** Scatter plots of observed and modelled CO_2 fluxes at 30-minute resolution at MU-OPD.
    e. **Figure S8.** Scatter plots of observed and modelled CO fluxes at 30-minute resolution at MU-OPD.
    f. **Figure S9.** Scatter plots of observed and modelled CH_4 fluxes at 30-minute resolution at MU-OPD.
    g. **Figure S10.** Scatter plots of observed and modelled CO_2 fluxes at 30-minute resolution at PR-RMV.
    h. **Figure S11.** Scatter plots of observed and modelled CO fluxes at 30-minute resolution at PR-RMV.
    i. **Figure S12.** Scatter plots of observed and modelled CH_4 fluxes at 30-minute resolution at PR-RMV.
    j. **Table S2.** Spearman correlation values between modelled and observed fluxes of CO_2, CO, and CH_4 at ZH-HAR, MU-OPD, and PR-RMV under different temporal and temperature conditions at 30-minute resolution.
5. (*page S22*) - Comparison of Decomposed Model Results to Observations
    a. **Figure S13.** Observed and modelled anthropogenic mean monthly diurnal CO_2 fluxes across cities.
    b. **Figure S14**. Observed and modelled anthropogenic mean monthly diurnal CO fluxes across cities.
    c. **Figure S15.** Observed and modelled anthropogenic mean monthly diurnal CH_4 fluxes across cities
6. (*page S26*) – Means and Standard Deviations of Emissions by Sector
    a. **Table S3.** Means ± standard deviations of calculated CO_2 fluxes (μmol m^{-2} s^{-1}) per month over one year per emission sector at each site.
    b. **Table S4.** Means ± standard deviations of calculated CO fluxes (μmol m^{-2} s^{-1}) per month over one year per emission sector at each site.
    c. **Table S5.** Means ± standard deviations of calculated CH_4 fluxes (μmol m^{-2} s^{-1}) per month over one year per emission sector at each site.
7. (*page S30*) – Diurnal Patterns of Modelled Anthropogenic Fluxes

a. **Figure S16.** Diurnal trends in Jul and Dec 2023 for important contributors to CO_2 fluxes across cities.
b. **Figure S17.** Diurnal trends in Jul and Dec 2023 for important contributors to CO fluxes across cities.
c. **Figure S18.** Diurnal trends in Jul and Dec 2023 for important contributors to CH_4 fluxes across cities.

8. (*page S32*) – Other Emissions
   a. **Figure S19.** Mean and standard deviations of monthly diurnal modelled 'other' fluxes at HAR of (a) CO_2 and (b) CO and CH_4.
9. (*page S33*) - References

Roughness Length and Displacement Height Derivation

Roughness length and displacement height maps for each site were developed in QGIS Desktop v.3.22.2 ('Białowieża') using the Morphology Calculator capability of the most updated version of the Urban Multi-scale Environmental Predictor (UMEP) For Processing add-on [1]. Roughness length and displacement height maps were developed for all sites at 100-m spatial resolution over the relevant footprint extents using the method from Macdonald et al. (1998) [2]. Inputs for UMEP include building and vegetation height maps, flux tower coordinates, wind direction sectors (for anisotropic estimates), and footprint extent (for area estimates). Anisotropic and area estimates were needed for the spin-up process in the FFP model [3] as described below. Because tree height data were not available in the study area of the RMV tower in Paris, a mean tree height was used in combination with tree location data. Footprints developed for this site should hence be used with caution.

In addition to the maps, first estimates of anisotropic z_0 and z_d were also derived using the UMEP-For-Processing add-on in QGIS using a 500-m radius around the tower at five-degree intervals from 0-360°. The maps and anisotropic estimates were implemented in a spin-up procedure to derive z_0 and z_d values unique to each footprint in each city.

The FFP spin-up process was as follows:

1) For each time step, the observed wind direction was used to select the anisotropic z_0 and z_d values from the corresponding five-degree interval.

2) These z_0 and z_d values were used to run the footprint model at 30-min temporal resolution.

3) Footprint-weighted z_0 and z_d values were computed from the 100-m spatial resolution maps.

4) These footprint-weighted z_0 and z_d derivations were used to determine footprints in the next iteration.

The spin-up process was repeated until the R^2 value between the weighted values computed in the iteration n and $n-1$ was nearly 1. For each city, three iterations were needed to achieve this outcome, providing confidence in the initial estimates.

Example Flux Footprint Visualization

In this section, an example of the flux footprint on 03 Jul 2023 at 11:30 UTC for each tower (Figure S1: Zurich-HAR, Figure S2: Munich-OPD, Figure S3: Paris-RMV) is provided. The coordinate system is in UTM. The white plus signs on each map denote their respective tower locations and the red contour lines depict the area that contributes 10-80% to the total footprint area in increments of 10%. The color scale refers to weight of the footprint (blue is a higher weight, yellow is lighter weight).

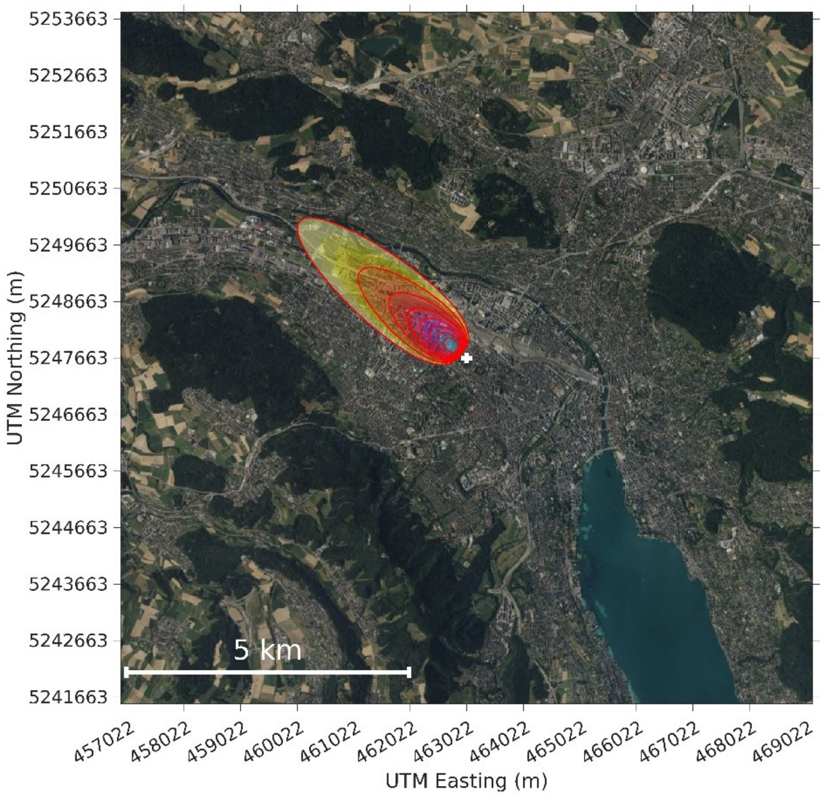

Figure S1. Example footprint at Zurich-HAR.

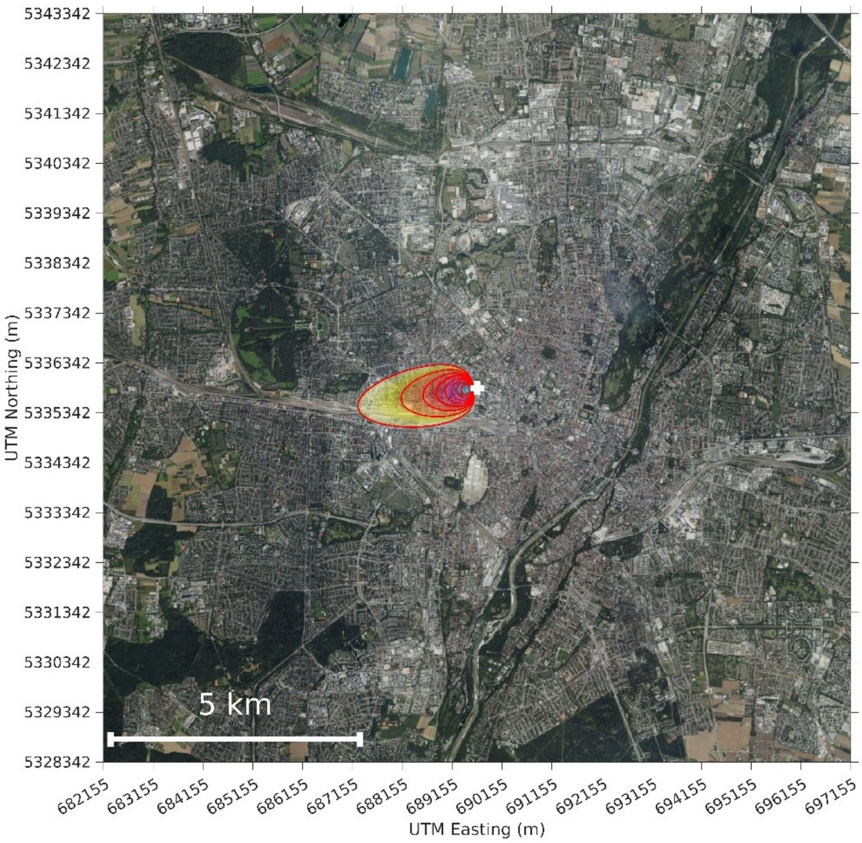

Figure S2. Example footprint at Munich-OPD.

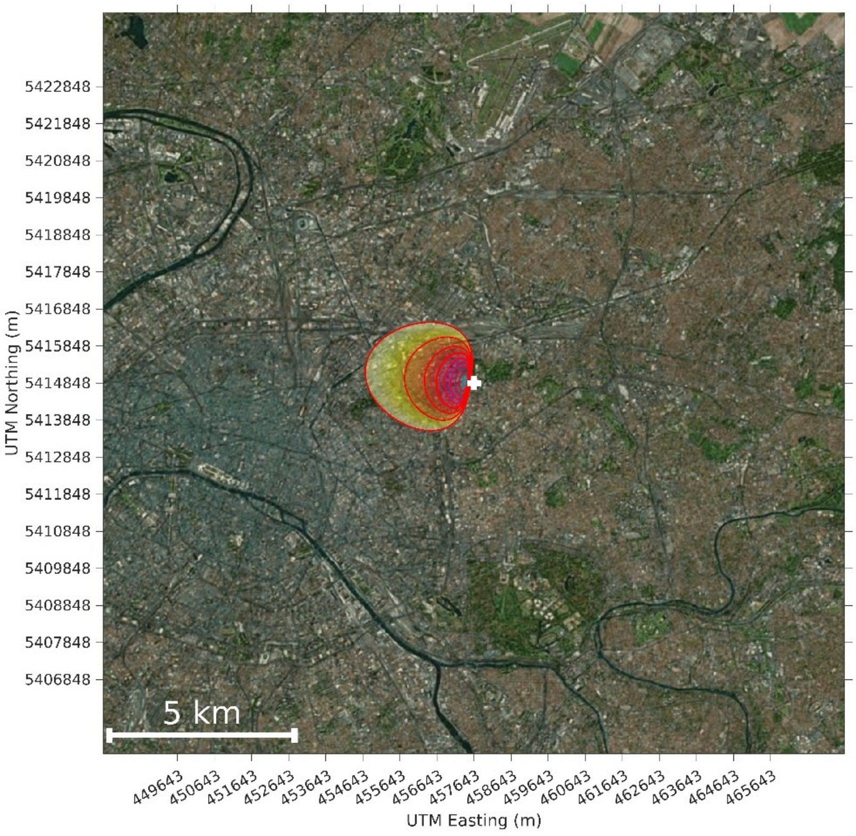

Figure S3. Example footprint at Paris-RMV.

TNO Emission Inventory Development

The Gridded Nomenclature for Reporting (GNFR) system is widely used in Europe to standardize emissions reporting. As mentioned in the main text, many different datasets are incorporated into emission inventory development as not all emission types and sources are closely monitored and therefore require proxy data. Table S1 below describes the letter code and definition of each GNFR sector and the proxy data used for each, if applicable. More details about emission inventory development for this study can be found in the main text as well as in Kuenen et al. (2022) [4], Super et al. (2020) [5], and Super et al. (2025) [6].

Table S1. List of GNFR sectors in the emission inventories and the proxy data used for downscaling the European emissions for Paris and Munich.

**Code**	**Sector**	**Proxy data**
A	Energy	Point sources + land use[1, 2, 3] (industry)
B	Industry	Point sources + land use[1, 2, 3] (industry)
C	Other stationary combustion	Built-up volume[4]
D	Fugitives	None
E	Solvents	None
F	Road transport	Vehicle km[5]
G	Shipping	Land use[1, 2, 3] (waterways)
H	Aviation	Land use[1, 2, 3] (airport)
I	Off-road transport	Land use[1, 2, 3] (agro-forest, industry, railroads), population[6]
J	Waste	None
K	Agriculture – livestock	Land use[1, 2, 3] (pastures)
L	Agriculture - other	Land use[1, 2, 3] (arable land)
O	Human respiration	Population[6]

[1]CLCplus Backbone land cover data (European Union's Copernicus Land Monitoring Service information, 2024) [7].
[2]Impervious Built-up data (European Union's Copernicus Land Monitoring Service information, 2020a) [8].
[3]CORINE Land Cover data (European Union's Copernicus Land Monitoring Service information, 2020b) [9].
[4]Copernicus Global Human Settlement Layer GHS-BUILT-V - R2023A built-up volume dataset [10].
[5]OpenTransportMap [11].
[6]Copernicus Global Human Settlement Layer GHS-POP - R2023A population volume dataset [12].

Correlations at 30-min Resolution

The main text focuses on comparisons between the observed and modelled fluxes of each species at all sites on a mean monthly diurnal scale in order to better assess how well the model captures overall temporal trends. In this section of the SI, similar comparisons are undertaken at 30-minute resolution to determine if the model performs well at high temporal resolution. Figures S4-S6 show the Hardau data for CO_2, CO, and CH_4 by 45° wind direction sector, respectively. Figures S7-9 and S10-S12 show the same for Oberpostdirektion and Romainville, respectively. Filled vs. unfilled points represent daytime vs. nighttime, and large vs. small points represent weekdays vs. weekends. All are color-coded by air temperature (°C). It should be noted that observed flux values greater than 1 µmol m^{-2} s^{-1} or less than -1 µmol m^{-2} s^{-1} were removed from all CO and CH_4 observational datasets to reduce the effect of outliers. The number of data points not shown in the graphs after post-processing are documented on each figure panel.

The highest correlations between the observed and modelled CO_2 fluxes at HAR occur from 45°-90° and 270°-315°, followed by 225°-270° and 315°-360°, all falling within the moderate range. The remaining wind sectors demonstrate poor correlation. Modelled fluxes tend to be higher at colder temperatures than at warmer temperatures, but this is not consistent across wind directions; however, the strongest correlation between the model and the observations with respect to temperature occurs at negative temperatures (Table S2). There does not appear to be any correlation with respect to time of day or day of week at 30-minute resolution, but Table S2 reveals moderate correlation during daytime hours on both weekdays and weekends; correlations during nighttime hours are consistently poor. Table S2 also separates wind direction into sectors of 10° to provide more information on where the better-modelled fluxes originate. It is not clear in the figure whether there is a pattern regarding when the model over- or under-estimates fluxes as compared to the observations.

Figure S5 indicates that the region with the highest Spearman correlation between modelled and observed CO fluxes is the area that is northeast of the Hardau tower (45°-90°), though the correlation is only moderate; of the eight wind direction sectors considered, three show poor correlation ($\rho < 0.38$). Modelled fluxes tend to be higher at colder temperatures than at warmer temperatures, possibly due to the role that temperature plays in determining the temporal profiles of some emission sectors. The number of data points makes it difficult to see any trends relating to the day of the week or hour of the day, but the results in Table S2 show that the correlation between modelled and observed fluxes is moderate for both weekdays and weekends and poor during both daytime and nighttime hours. It is also moderate at most temperatures, with the exception of those above 30°C, which indicates poor correlation.

In Figure S6, it is extremely apparent that there is almost no correlation between the modelled and observed CH_4 fluxes at Hardau in any given wind sector. Panels (a) and (b) show that the modelled fluxes are higher at colder temperatures, like with CO; because the temporal profiles applied to both species were the same, any effect of temperature that was considered would naturally show itself in the modelled CH_4 as well. There is no clear pattern between daytime vs. nighttime or weekday vs. weekend correlations in this figure, and Table S2 shows that the correlations are often slightly negative. All correlations, regardless of time of day, wind direction, or air temperature, are considered to be poor.

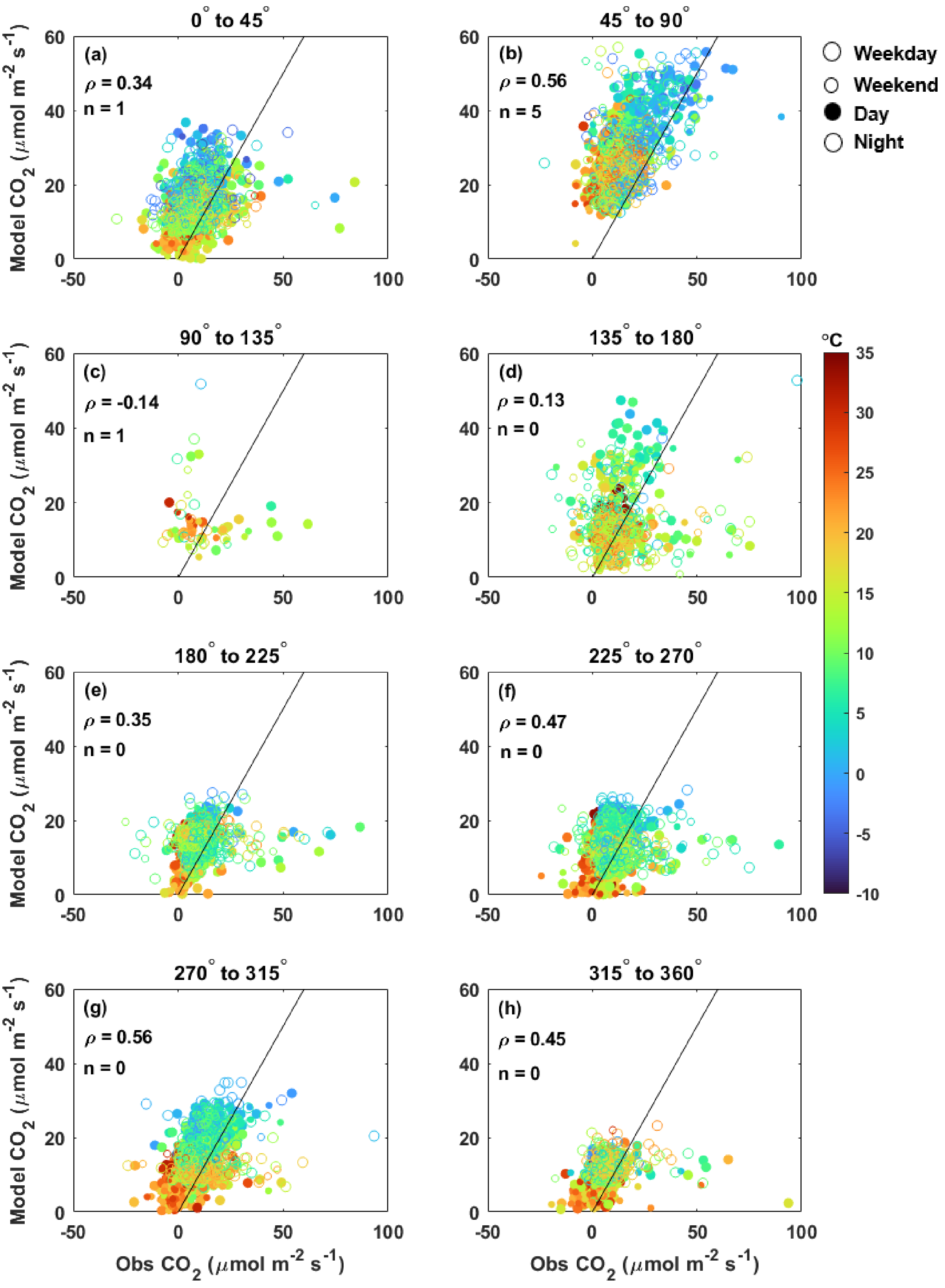

Figure S4. Scatter plots of observed (x-axis) and modelled (y-axis) CO_2 fluxes at 30-minute resolution at ZH-HAR separated by wind direction and color-coded by air temperature in °C. Filled = daytime fluxes; empty = nighttime fluxes; large = weekday fluxes; small = weekend fluxes. The black line is the 1:1 line. The Spearman correlation coefficient and number of outliers for each wind sector is provided on its respective panel.

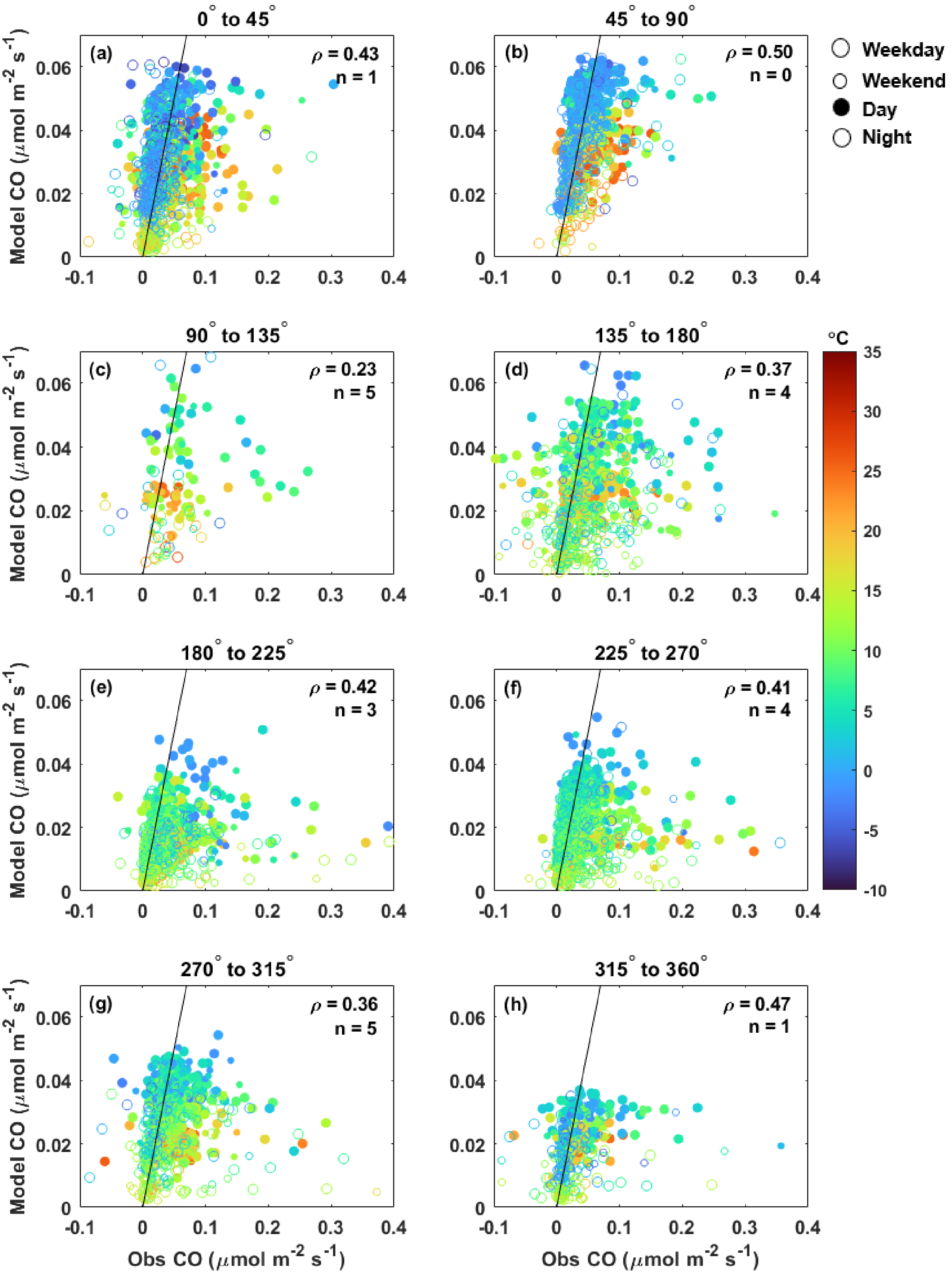

Figure S5. Scatter plots of observed (x-axis) and modelled (y-axis) CO fluxes at 30-minute resolution at ZH-HAR separated by wind direction and color-coded by air temperature in °C. Filled = daytime fluxes; empty = nighttime fluxes; large = weekday fluxes; small = weekend fluxes. The black line is the 1:1 line. The Spearman correlation coefficient and number of outliers for each wind sector is provided on its respective panel.

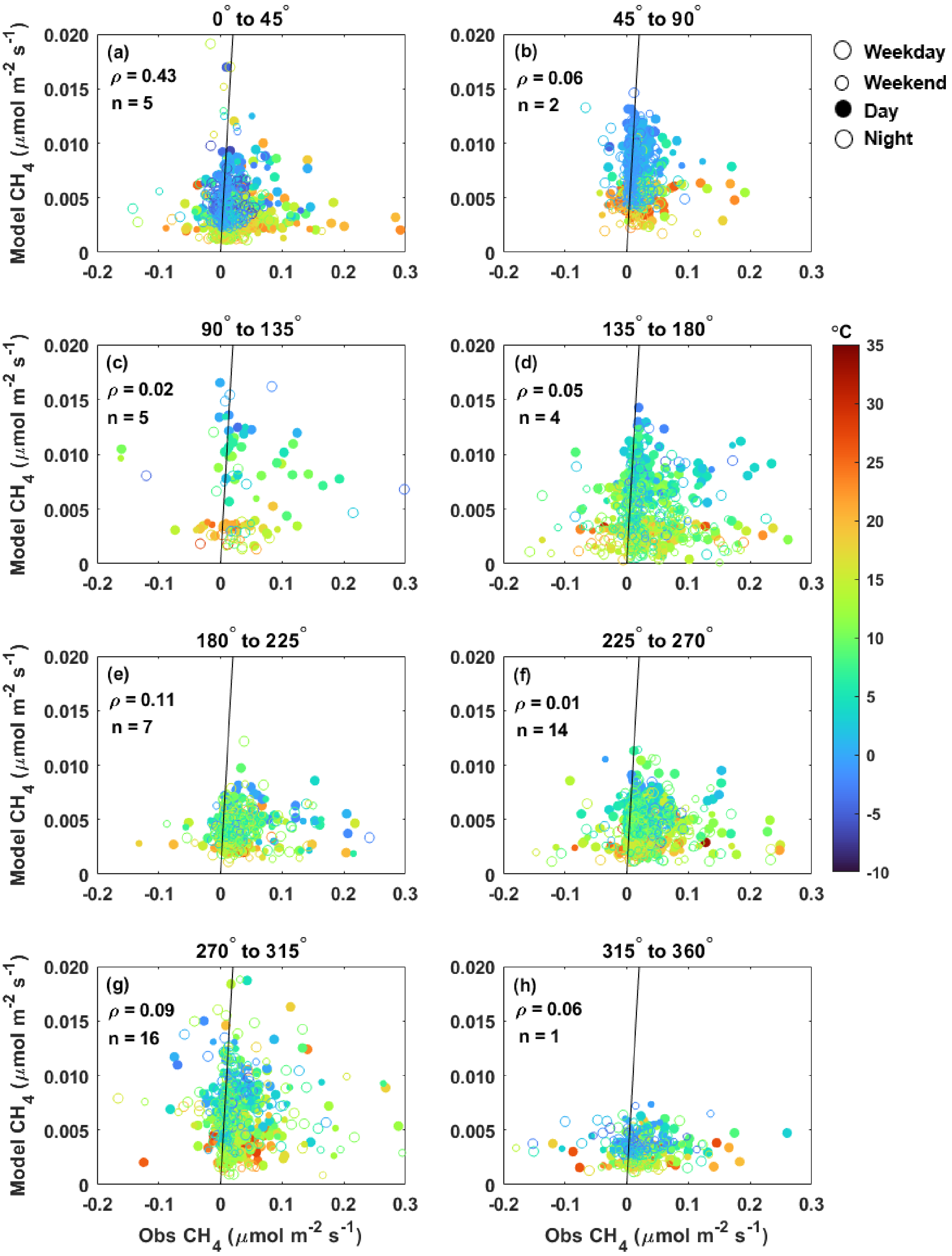

Figure S6. Scatter plots of observed (x-axis) and modelled (y-axis) CH_4 fluxes at 30-minute resolution at ZH-HAR separated by wind direction and color-coded by air temperature in °C. Filled = daytime fluxes; empty = nighttime fluxes; large = weekday fluxes; small = weekend fluxes. The black line is the 1:1 line. The Spearman correlation coefficient and number of outliers for each wind sector is provided on its respective panel.

The strongest correlations for CO_2 at OPD occur from 270°-315°, followed by 225°-270° and 315°-360° (all moderate) while poor correlation is observed in the remaining wind direction sectors. A seasonal trend appears here again with higher fluxes in colder temperatures and lower fluxes in warmer temperatures, but the correlations between the model and observations vary from weakly negative to moderate at different temperatures. Correlations during daytime hours and weekdays are moderate while correlations during nighttime hours and on weekends are weak. Full campaign performance is weaker at OPD than at HAR. Here, there is also no clear trend regarding when the model over- or under-estimates the observed fluxes.

Figure S8 indicates that the emission inventory for Munich performs better than that for Zurich with respect to CO, as three wind direction sectors (45°-90°, 180°-225°, and 225°-270°) display moderate correlation. Despite the removal of outliers, there are still many observations at OPD, often at higher air temperatures, that are much higher than modelled values or are negative; this likely impacts the correlation analysis. There is no clear pattern with respect to weekday vs. weekend or hour of day, although Table S2 indicates that correlations for weekdays and nighttime hours are moderate while correlations for weekends or daytime hours are poor. Table S2 also indicates that correlations between the modelled and observed fluxes are moderate at temperatures up to 20°C, above which the correlations are considered to be poor.

Figure S9 demonstrates that the Munich emission inventory severely underperforms with respect to CH_4, as seen in the Zurich emission inventory. Correlations at all wind directions are poor or even negative (Table S2), except at 0°-45°, which only just meets the threshold for moderate correlation. There is also no correlation at any time of day, day of week, or air temperature, with the exception of $T \geq 30$°C; surprisingly, the correlation for this temperature range was good ($\rho = 0.78$). While some improvements still need to be made, it appears that the Munich inventory performance at 30-minute resolution does surpass that of the Zurich emission inventory for the study areas of their respective EC towers.

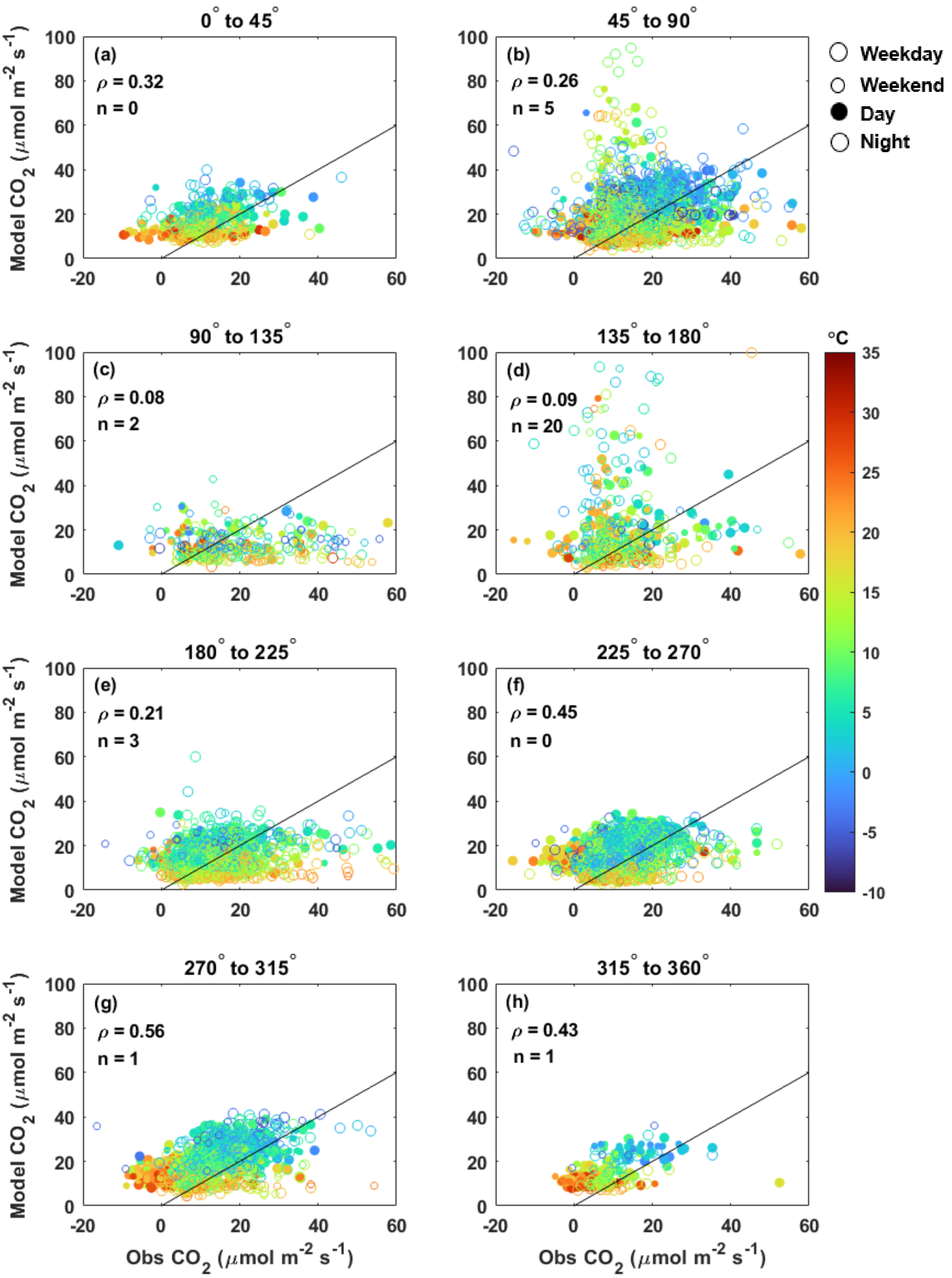

Figure S7. Scatter plots of observed (x-axis) and modelled (y-axis) CO_2 fluxes at 30-minute resolution at MU-OPD separated by wind direction and color-coded by air temperature in °C. Filled = daytime fluxes; empty = nighttime fluxes; large = weekday fluxes; small = weekend fluxes. The black line is the 1:1 line. The Spearman correlation coefficient and number of outliers for each wind sector is provided on its respective panel.

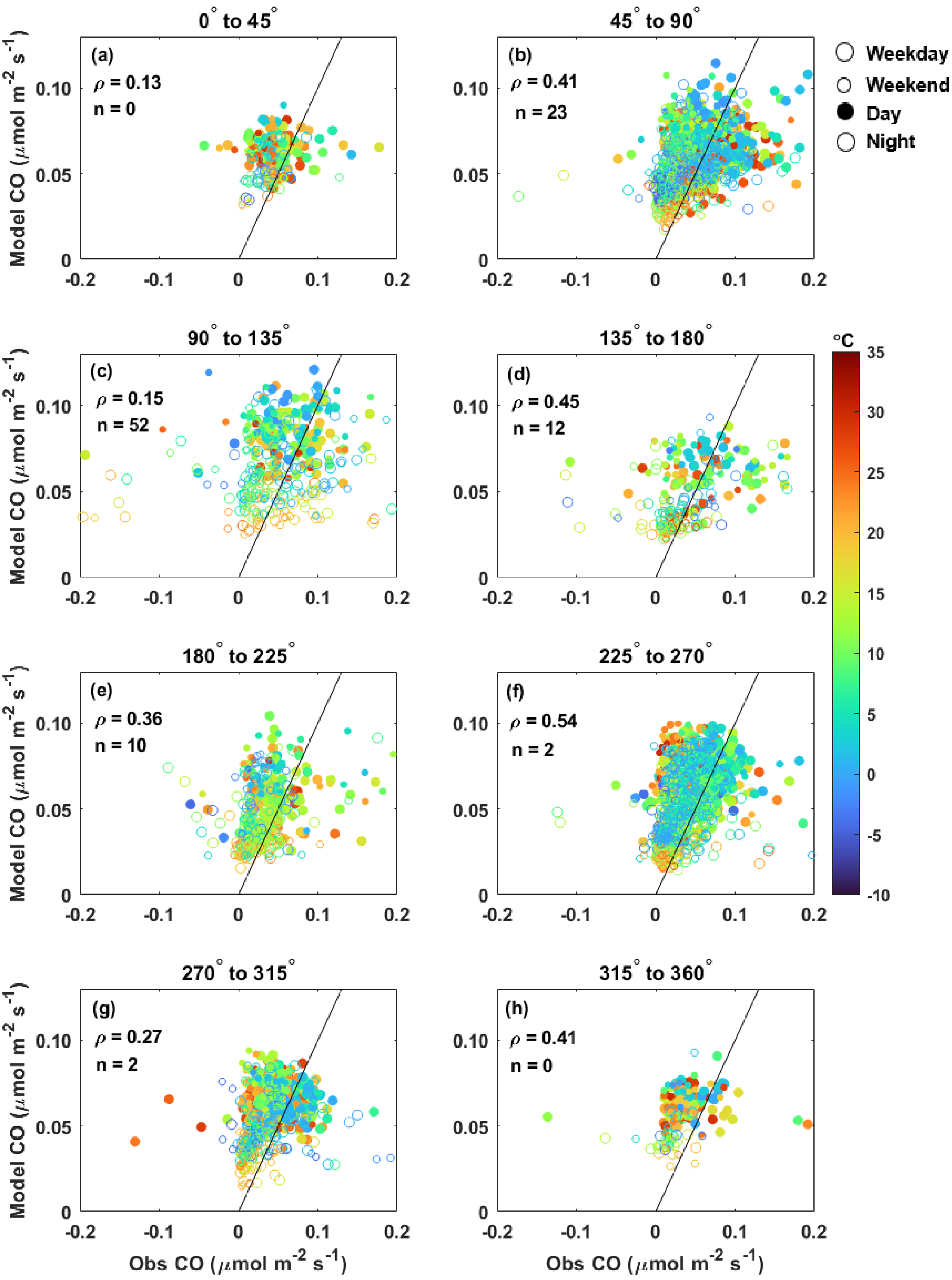

Figure S8. Scatter plots of observed (x-axis) and modelled (y-axis) CO fluxes at 30-minute resolution at MU-OPD separated by wind direction and color-coded by air temperature in °C. Filled = daytime fluxes; empty = nighttime fluxes; large = weekday fluxes; small = weekend fluxes. The black line is the 1:1 line. The Spearman correlation coefficient and number of outliers for each wind sector is provided on its respective panel.

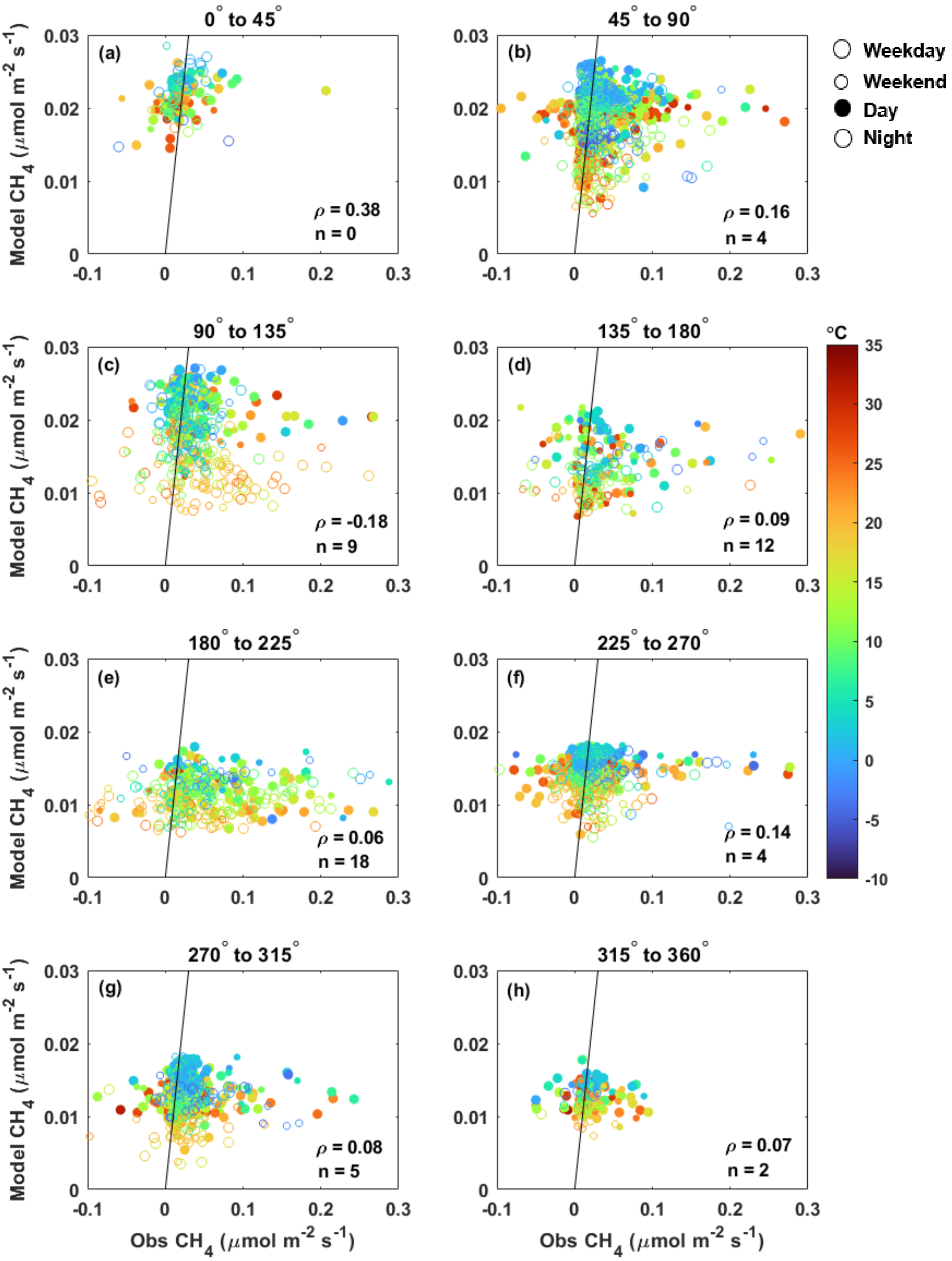

Figure S9. Scatter plots of observed (x-axis) and modelled (y-axis) CH_4 fluxes at 30-minute resolution at MU-OPD separated by wind direction and color-coded by air temperature in °C. Filled = daytime fluxes; empty = nighttime fluxes; large = weekday fluxes; small = weekend fluxes. The black line is the 1:1 line. The Spearman correlation coefficient and number of outliers for each wind sector is provided on its respective panel.

Figure S10 shows that the best correlation between modelled and observed CO_2 fluxes at RMV occurs from 225°-315°, followed by 180°-225° (all moderate); remaining wind direction sectors have poor correlation values. The model appears to consistently overestimate fluxes with respect to the observations, and the same trend with respect to temperature is observed and RMV as at both HAR and OPD (though it should be noted that the temperature range considered is smaller); correlations are moderate from 5°C-15°C and poor for remaining temperatures. Full-campaign correlations show that the model performs better at RMV than at the other two sites. There is no difference in correlation between weekdays and weekends. Lastly, the same trend of improved correlation during daytime hours vs. nighttime hours is also observed at this site.

Similarly to Figure S8, Figure S11 demonstrates that the modelled fluxes derived from the emission inventory for Paris are moderately correlated with CO observed fluxes for most wind directions, with the exception of 0°-45°, 90°-135°, and 315°-360°. There does not appear to be any pattern for any wind direction sector with regards to air temperature, time of day, or day of week, though most correlations found in Table S2 tend to be moderate with the exception of the poor correlation below 0°C and the strong correlation ($\rho = 0.69$) from 20°C-25°C. The use of default temporal scaling factors might need to be revisited, as they are meant for all of Europe and therefore cannot represent the behaviors and conditions unique to Paris. What is interesting here is that the model overestimates CO fluxes with respect to the observations, which could either mean that the inventory itself is overestimating CO emissions in the area around the RMV tower or that the MGA-7 did not capture the fluxes in the region well. Performance of the model with respect to observations varies by 10° wind direction sector (Table S2), ranging from weakly negative to moderately positive.

Lastly, Figure S12 indicates poor performance of modelled CH_4 fluxes with respect to observations. The poor correlation persists with air temperature, time of day, and day of week (Table S2), as well as with each of the 10° wind direction sectors.

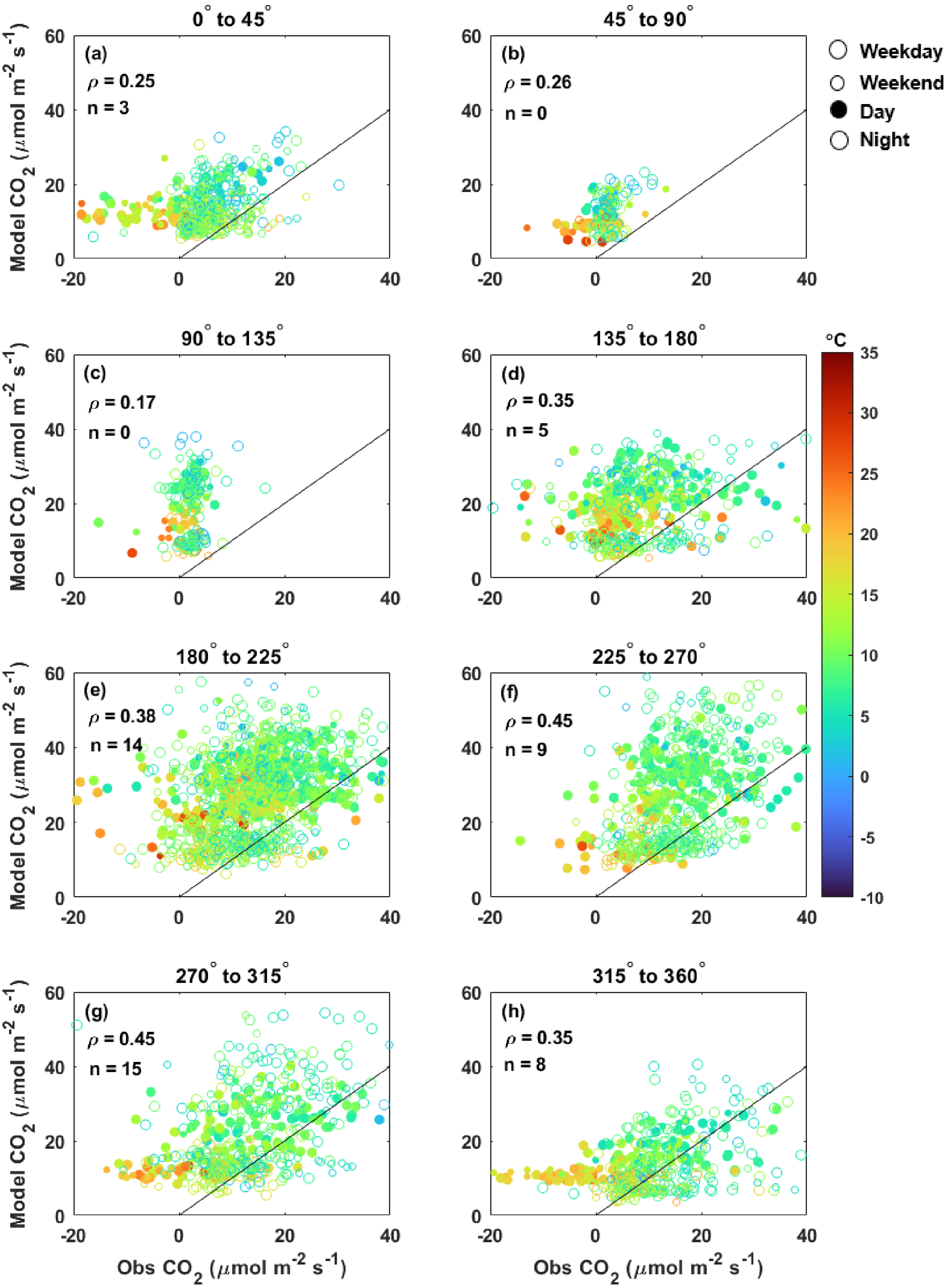

Figure S10. Scatter plots of observed (x-axis) and modelled (y-axis) CO_2 fluxes at 30-minute resolution at PR-RMV separated by wind direction and color-coded by air temperature in °C. Filled = daytime fluxes; empty = nighttime fluxes; large = weekday fluxes; small = weekend fluxes. The black line is the 1:1 line. The Spearman correlation coefficient and number of outliers for each wind sector is provided on its respective panel.

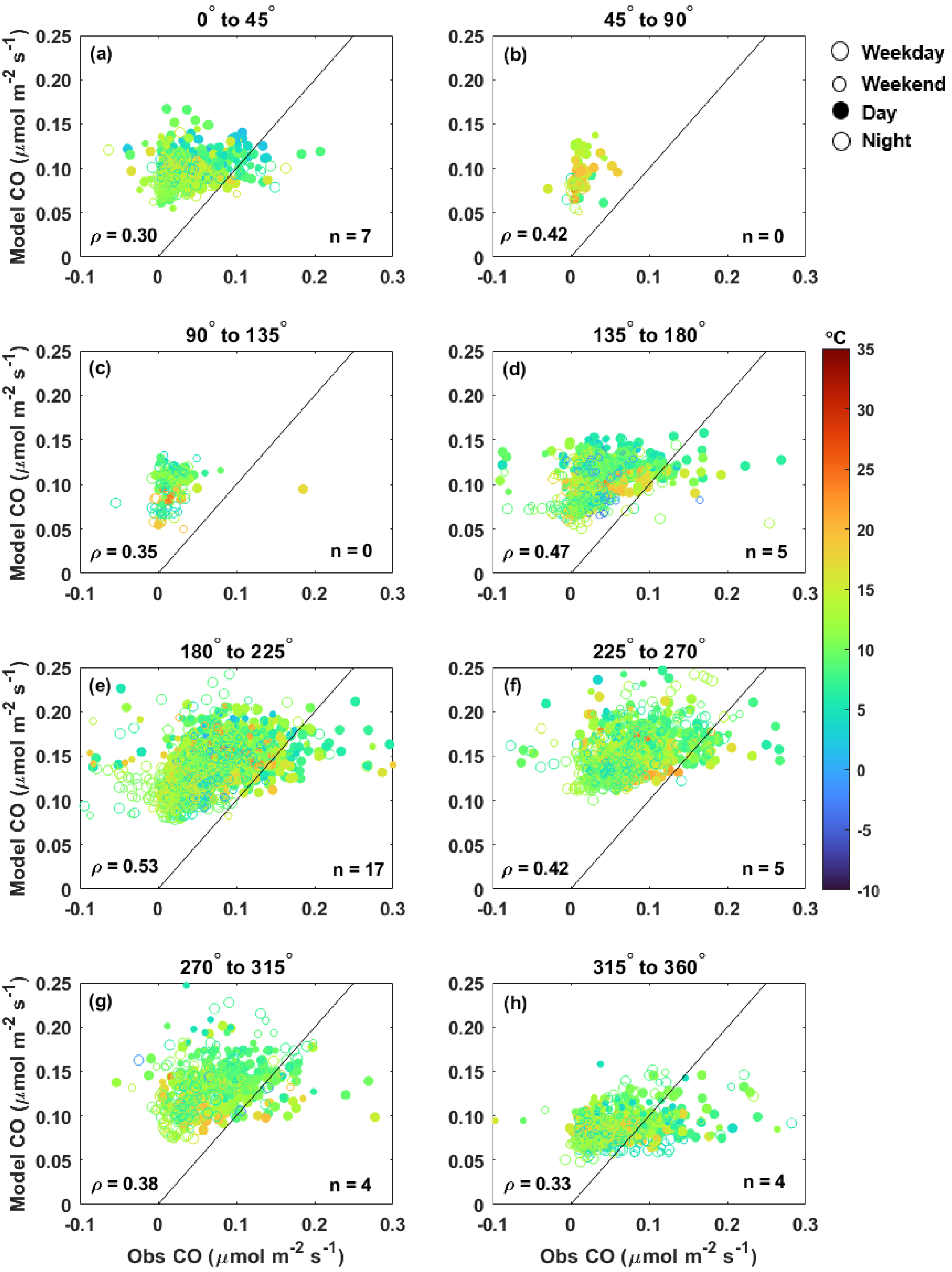

Figure S11. Scatter plots of observed (x-axis) and modelled (y-axis) CO fluxes at 30-minute resolution at PR-RMV separated by wind direction and color-coded by air temperature in °C. Filled = daytime fluxes; empty = nighttime fluxes; large = weekday fluxes; small = weekend fluxes. The black line is the 1:1 line. The Spearman correlation coefficient and number of outliers for each wind sector is provided on its respective panel.

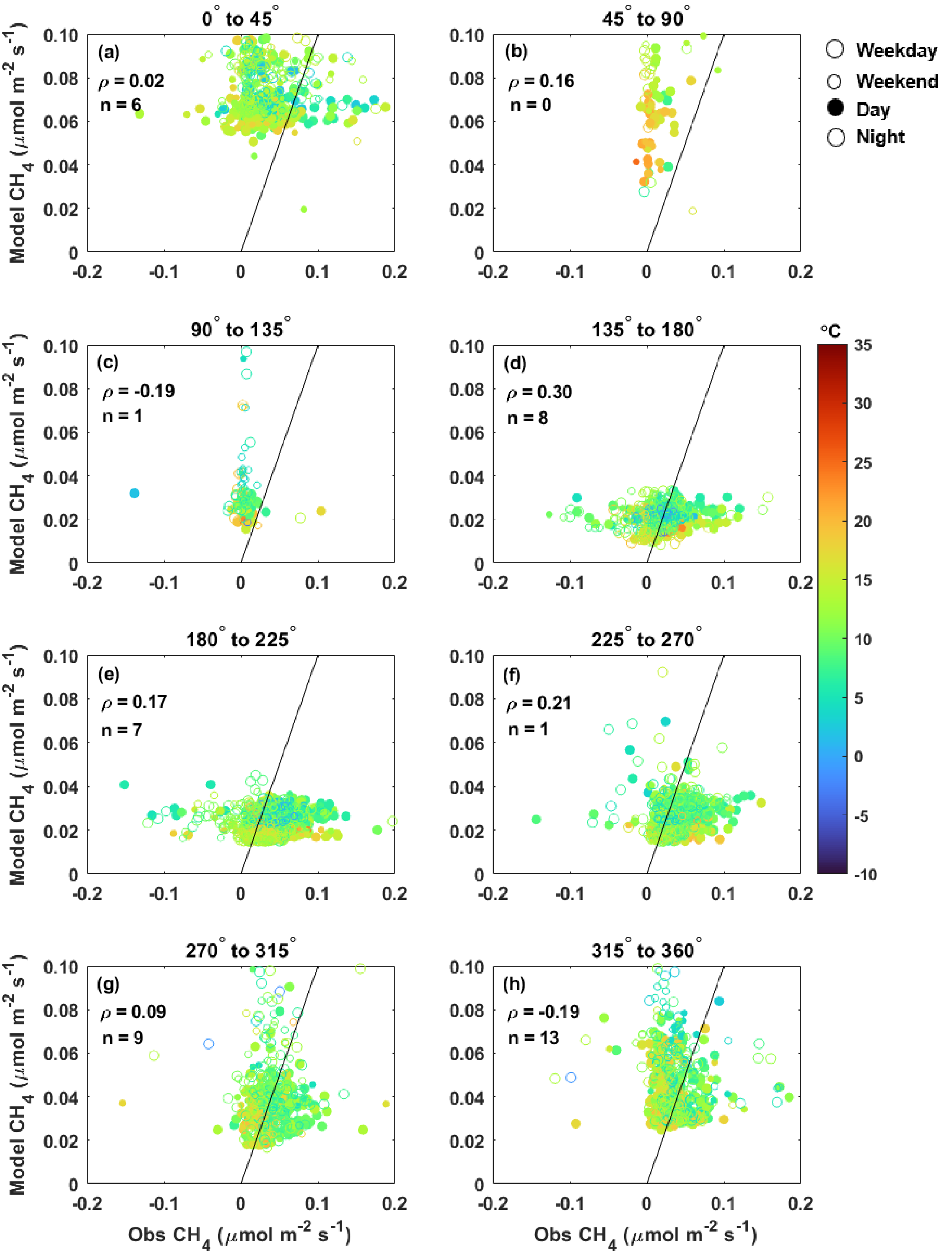

Figure S12. Scatter plots of observed (x-axis) and modelled (y-axis) CH_4 fluxes at 30-minute resolution at PR-RMV separated by wind direction and color-coded by air temperature in °C. Filled = daytime fluxes; empty = nighttime fluxes; large = weekday fluxes; small = weekend fluxes. The black line is the 1:1 line. The Spearman correlation coefficient and number of outliers for each wind sector is provided on its respective panel.

Overall, the Paris emission inventory appears to perform best for CO_2 and CO, followed by Zurich and Munich. The lower performance during nighttime hours may stem from the boundary layer height being too low and thereby affecting either the flux observations or the footprint model (or both); interestingly, the model performs better at nighttime hours with respect to CO at all three sites. The Munich and Paris inventories both perform better for CH_4 than the Zurich inventory, but all show extremely weak correlation between the modelled and observed fluxes. Table S2, below, provides more details regarding model and observation correlations at high temporal resolution under different circumstances. There is generally not much difference between weekday and weekend correlations at any site for any species, except for CO_2 and CO at Munich.

Table S2. Spearman correlation values between modelled and observed fluxes of CO_2, CO, and CH_4 at HAR, OPD, and RMV under different temporal and temperature conditions at 30-minute resolution. Bolded values indicate 'good' correlation ($\rho \geq 0.68$). Dashes indicate 'no data'.

Parameter	HAR			OPD			RMV		
	CO_2	CO	CH_4	CO_2	CO	CH_4	CO_2	CO	CH_4
Full Campaign	0.45	0.43	0.05	0.36	0.41	0.10	0.48	0.51	0.10
Weekday	0.46	0.43	0.03	0.40	0.48	0.10	0.49	0.50	0.10
Weekend	0.41	0.42	0.10	0.26	0.32	0.11	0.46	0.52	0.10
Daytime	0.56	0.27	0.05	0.46	0.10	0.15	0.63	0.42	0.03
Nighttime	0.30	0.36	0.04	0.34	0.40	0.03	0.44	0.52	0.20
Weekday_Day	0.56	0.22	0.01	0.48	0.19	0.16	0.61	0.42	0.04
Weekday_Night	0.33	0.37	0.03	0.37	0.48	0.01	0.45	0.51	0.22
Weekend_Day	0.56	0.31	0.12	0.45	0.07	0.13	0.67	0.45	0.04
Weekend_Night	0.21	0.33	0.07	0.24	0.23	0.07	0.41	0.53	0.19
T < 0°C	0.60	0.52	0.13	0.36	0.41	-0.08	-	0.01	0.32
0°C ≤ T < 5°C	0.46	0.52	0.22	0.53	0.47	0.15	0.34	0.45	0.07
5°C ≤ T < 10°C	0.31	0.39	0.05	0.49	0.41	0.12	0.42	0.49	0.18
10°C ≤ T < 15°C	0.42	0.46	0.08	0.11	0.42	0.04	0.50	0.51	0.03
15°C ≤ T < 20°C	0.40	0.54	0.11	0.00	0.47	0.08	0.30	0.37	-0.03
20°C ≤ T < 25°C	0.52	0.40	0.06	-0.17	0.34	0.01	0.28	**0.69**	-0.12
25°C ≤ T < 30°C	0.55	0.46	-0.15	-0.11	0.05	0.18	0.24	-	-
T ≥ 30°C	0.13	0.27	0.34	0.44	0.25	**0.78**	-	-	-
0° ≤ WD < 10°	0.41	0.39	0.07	-	-	-	0.15	0.41	0.01
10° ≤ WD < 20°	0.38	0.39	0.05	-	-	-	0.13	0.45	0.18
20° ≤ WD < 30°	0.39	0.39	-0.06	0.52	0.17	0.23	0.37	0.28	0.07
30° ≤ WD < 40°	0.28	0.44	0.02	0.25	0.13	0.39	0.22	0.19	0.01
40° ≤ WD < 50°	0.48	0.56	0.04	0.36	0.25	0.25	0.17	0.24	-0.01
50° ≤ WD < 60°	0.62	0.49	0.08	0.33	0.36	0.23	0.29	0.64	0.23
60° ≤ WD < 70°	0.49	0.43	-0.02	0.22	0.52	0.27	0.35	-0.09	0.03
70° ≤ WD < 80°	-	-	-	0.30	0.44	0.02	-	-	-
80° ≤ WD < 90°	-	-	-	0.15	0.47	0.16	-	-	-
90° ≤ WD < 100°	-	-	-	0.10	0.22	0.11	-	-	-
100° ≤ WD < 110°	-	-	-	0.01	-0.01	-0.01	0.21	0.39	-0.01
110° ≤ WD < 120°	0.10	0.24	0.11	0.12	0.18	-0.27	0.15	0.33	-0.04
120° ≤ WD < 130°	-0.30	-0.05	-0.11	0.23	0.23	0.02	0.19	0.32	-0.32
130° ≤ WD < 140°	0.09	0.35	-0.12	0.10	0.19	-0.15	-0.05	0.33	0.07
140° ≤ WD < 150°	-0.08	0.18	-0.03	0.18	0.31	0.02	0.20	0.23	0.15
150° ≤ WD < 160°	0.31	0.50	0.20	0.29	0.47	0.10	0.35	0.45	0.34
160° ≤ WD < 170°	0.14	0.43	0.06	0.02	0.50	0.21	0.43	0.52	0.12
170° ≤ WD < 180°	0.28	0.43	-0.03	0.09	0.42	0.14	0.37	0.56	0.08
180° ≤ WD < 190°	0.26	0.41	0.08	0.07	0.29	-0.02	0.43	0.55	0.16

190° ≤ WD < 200°	0.33	0.24	-0.22	0.20	0.28	0.29	0.32	0.56	0.14
200° ≤ WD < 210°	0.22	0.55	0.22	0.17	0.39	-0.04	0.40	0.51	0.14
210° ≤ WD < 220°	0.33	0.51	0.18	0.38	0.44	0.21	0.44	0.43	0.32
220° ≤ WD < 230°	0.40	0.49	0.29	0.26	0.29	-0.10	0.37	0.45	0.30
230° ≤ WD < 240°	0.48	0.57	0.10	0.36	0.56	0.33	0.42	0.34	0.10
240° ≤ WD < 250°	0.45	0.41	0.00	0.40	0.49	0.08	0.48	0.41	0.12
250° ≤ WD < 260°	0.48	0.33	0.06	0.53	0.54	0.19	0.33	0.41	0.17
260° ≤ WD < 270°	0.52	0.29	-0.24	0.51	0.55	0.23	0.48	0.44	0.31
270° ≤ WD < 280°	0.65	0.43	-0.02	0.61	0.27	0.11	0.59	0.38	0.26
280° ≤ WD < 290°	0.56	0.40	0.10	0.60	0.39	0.20	0.40	0.39	0.04
290° ≤ WD < 300°	0.54	0.32	0.10	0.50	0.22	-0.04	0.50	0.47	0.24
300° ≤ WD < 310°	0.54	0.23	0.04	0.36	0.04	0.00	0.43	0.43	0.13
310° ≤ WD < 320°	0.43	0.32	0.01	0.40	0.34	0.22	0.52	0.56	0.31
320° ≤ WD < 330°	0.43	0.54	0.13	0.41	0.21	0.07	0.59	0.50	0.23
330° ≤ WD < 340°	0.44	0.39	-0.08	0.37	0.61	0.10	0.32	0.22	-0.09
340° ≤ WD < 350°	0.39	0.44	0.02	-	-	-	0.15	0.15	-0.13
350° ≤ WD < 360°	0.49	0.37	-0.12	-	-	-	0.18	0.35	-0.16

Comparison of Decomposed Model Results to Observations

Figure 1 in the main text indicates that the sum of the modelled anthropogenic CO_2 fluxes across emission sectors is, on average, higher than the observations (especially at OPD and RMV), and that this discrepancy is not fully explained by the inclusion of biogenic CO_2 fluxes. While additional work needs to be done to assess how to close the gap between the models and the observations, there is still enough information to offer a starting point regarding the prioritization of emission sectors either to target in reduction plans or to revisit in inventory development.

Figure S13 compares mean monthly diurnal CO_2 flux observations and model results by emission sector across the full measurement campaign (Table 1) in (a) HAR, (b) OPD, and (c) RMV. As shown previously, stationary combustion dominates the signal throughout the campaign at all three sites. While it is difficult to discern a diurnal pattern in the observations, there is a clear seasonal trend that appears to follow that of the stationary combustion profile. This can be interpreted in one of two ways: 1) stationary combustion should be responsible for most of the signal at the tower and the other emission sectors are overestimated, or 2) stationary combustion itself is overestimated in the area around the tower. Either way, this sector is clearly the first that should be addressed in any reduction plan, and the benefits can be optimized by hour of day and month of year. Other emission sectors exhibiting a seasonal pattern, either because of human activity throughout the year or because of temperature dependence, should be further examined within the inventory at HAR and RMV as well in the area of the footprints at each site. The seasonal trend in observations is less evident at OPD, highlighting the need to look into more detail at emissions from sectors that do not exhibit a seasonal pattern when developing reduction plans. Figures S14 and S15 compare results across cities for CO fluxes and CH_4 fluxes, respectively, and are discussed in the main paper with Figure 2.

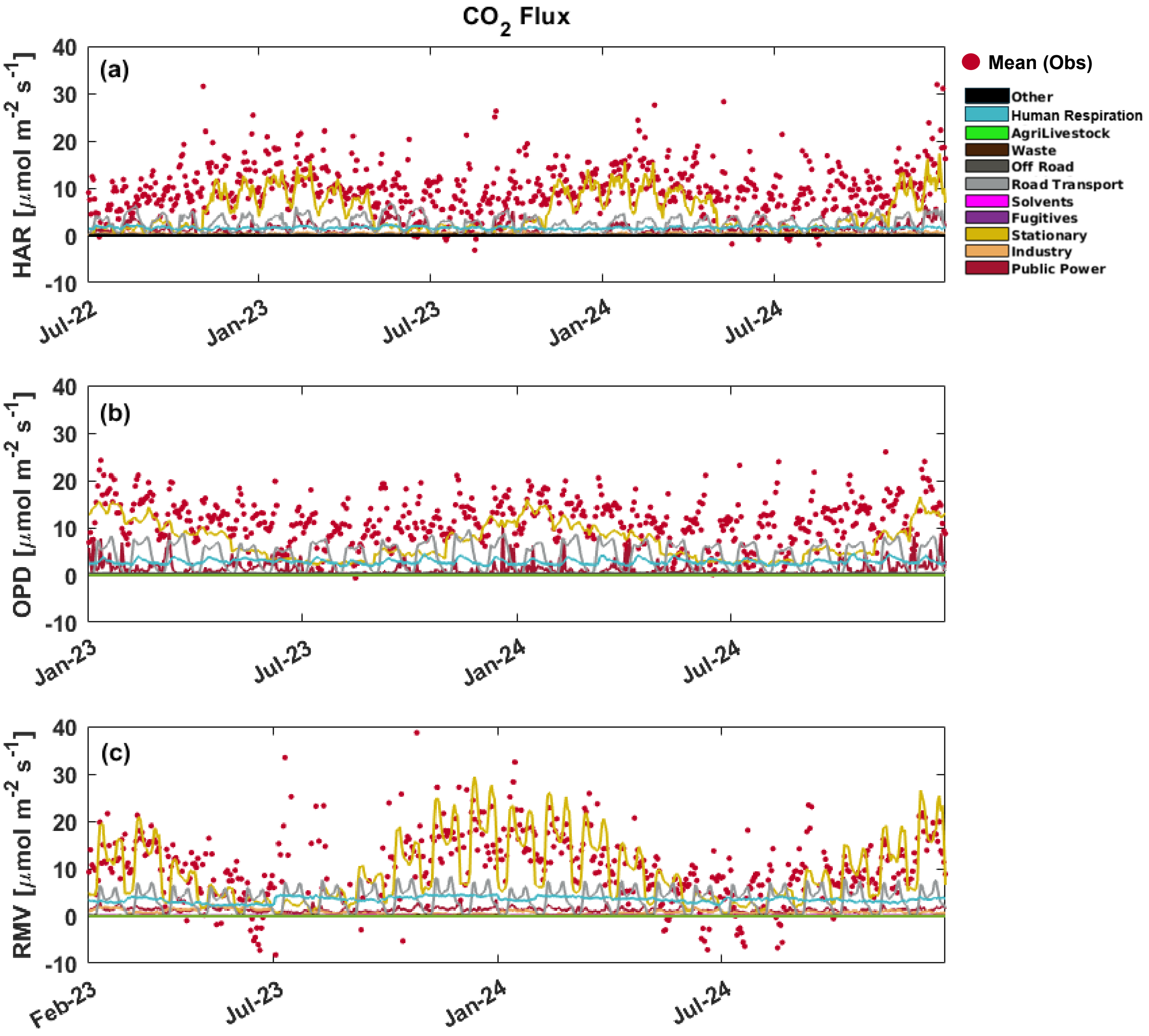

Figure S13. Observed (red dots) and modelled anthropogenic (lines) mean monthly diurnal CO_2 fluxes at (a) HAR, (b) OPD, and (c) RMV.

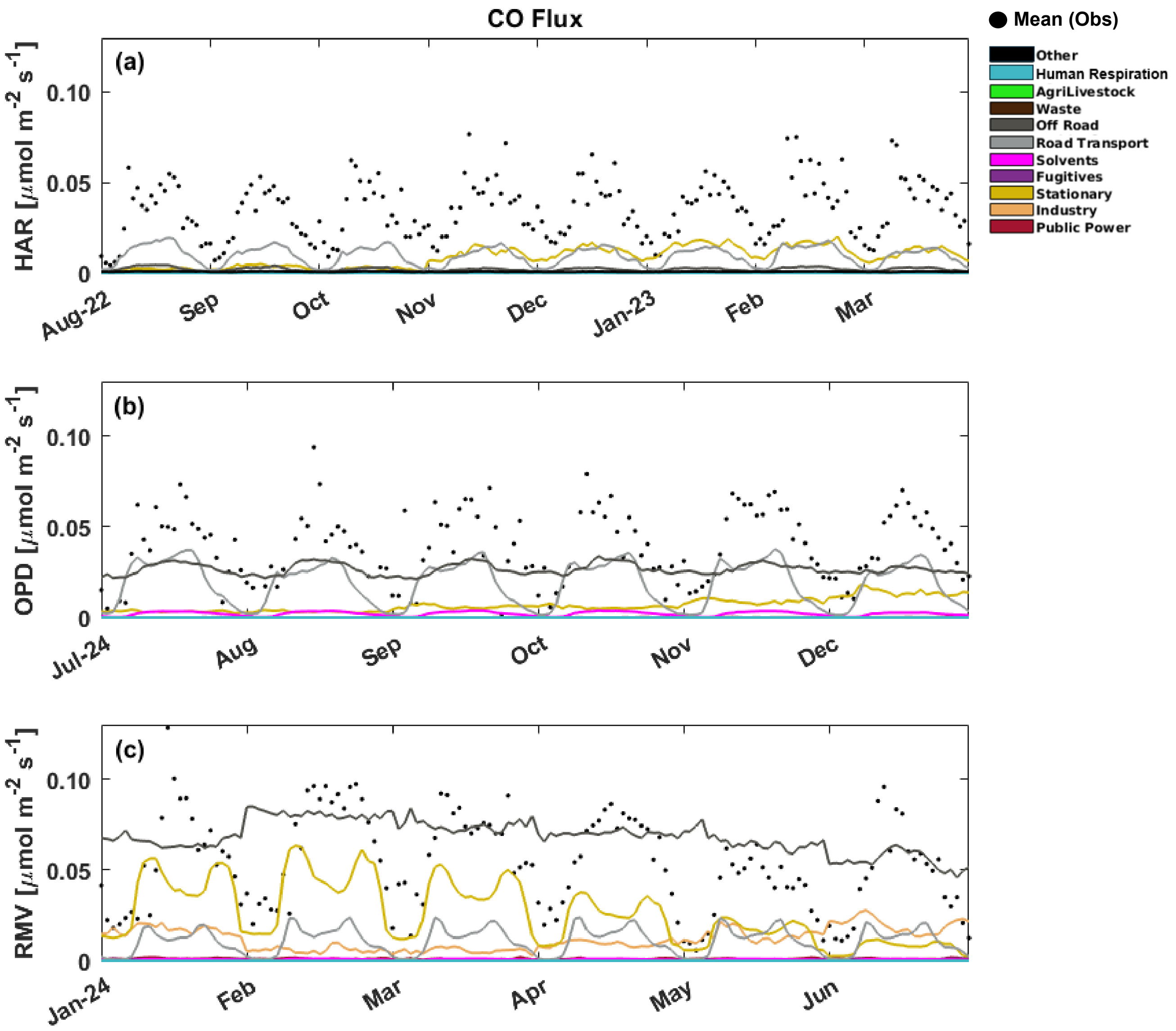

Figure S14. Observed (black dots) and modelled anthropogenic (lines) mean monthly diurnal CO fluxes at (a) HAR, (b) OPD, and (c) RMV.

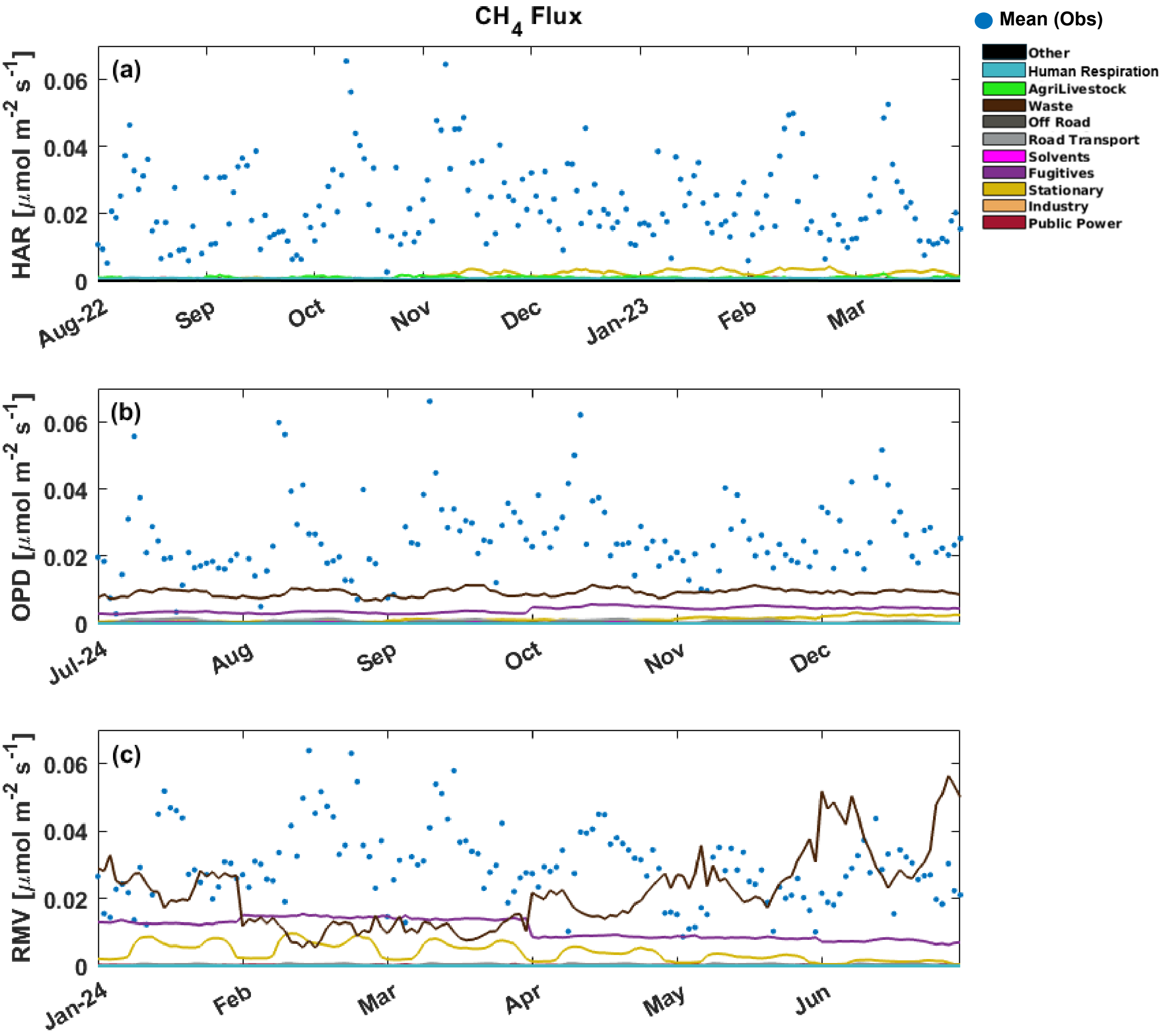

Figure S15. Observed (blue dots) and modelled anthropogenic (lines) mean monthly diurnal CH_4 fluxes at (a) HAR, (b) OPD, and (c) RMV.

Means and Standard Deviations of Emissions by Sector

As explained in Section 3.2.1 and Figure 3 in the main text, some emission sectors display seasonal patterns in the monthly average fluxes for each species while others appear to remain constant throughout the calendar year. However, with sectors such as stationary combustion dominating the signal at the flux towers, the patterns of other contributors to the total flux signal can be missed. Tables S3-5 provide the means and standard deviations of the contribution of each emission sector (A-R, where R is ‘other’) for each month examined in Figure 3 of the main text (Mar 2023-Feb 2024) for CO_2, CO, and CH_4, respectively. Each grid cell contains three rows representing HAR, OPD, and RMV, in that order. Emission sectors that did not contribute to the signal (i.e. had both means and standard deviations of or near 0 µmol m^{-2} s^{-1}) for any of the sites were excluded; sectors within this category varied by species. Standard deviations of or near 0 µmol m^{-2} s^{-1} were omitted in each grid cell where applicable.

Table S3. Means ± standard deviations of calculated CO_2 fluxes (μmol m^{-2} s^{-1}) per month over one year per GNFR emission sector at each site. See Table S1 for information on GNFR emission sectors and their abbreviations.

	A	B	C	D	E	F	G	I	J	O	R
**Mar**	**HAR:** 0.59 ± 2.7<br>**OPD:** 1.79 ± 11.0<br>**RMV:** 1.53 ± 1.66	0.38 ± 0.62<br>0<br>0.71 ± 1.18	8.4 ± 6.5<br>9.2 ± 4.3<br>13.5 ± 7.2	0<br>0.25 ± 0.064<br>0.013 ± 0.019	0 ± 0.001<br>0.19 ± 0.135<br>0.123 ± 0.079	2.5 ± 2.3<br>4.9 ± 3.5<br>3.8 ± 2.5	0.001 ± 0.004<br>0<br>0.001 ± 0.002	0.183 ± 0.110<br>0.35 ± 0.095<br>0.095 ± 0.025	0.05 ± 0.25<br>0<br>0.058 ± 0.149	1.54 ± 0.66<br>2.8 ± 1.00<br>3.8 ± 1.32	0.009 ± 0.004<br>0<br>0
**Apr**	**HAR:** 0.189 ± 4.3<br>**OPD:** 1.03 ± 3.5<br>**RMV:** 1.13 ± 1.38	0.49 ± 0.82<br>0<br>1.38 ± 1.32	6.6 ± 5.0<br>8.8 ± 4.4<br>7.7 ± 5.2	0<br>0.20 ± 0.053<br>0.017 ± 0.013	0 ± 0.001<br>0.22 ± 0.146<br>0.097 ± 0.068	3.3 ± 2.8<br>4.1 ± 3.1<br>3.3 ± 2.3	0.002 ± 0.014<br>0<br>0.001 ± 0.002	0.20 ± 0.121<br>0.37 ± 0.097<br>0.077 ± 0.032	0.076 ± 0.37<br>0<br>0.019 ± 0.142	1.76 ± 0.64<br>3.0 ± 0.97<br>2.8 ± 1.54	0.010 ± 0.003<br>0<br>0
**May**	**HAR:** 3.1 ± 5.1<br>**OPD:** 1.92 ± 5.8<br>**RMV:** 1.63 ± 1.44	0.45 ± 0.72<br>0<br>1.88 ± 1.20	1.77 ± 1.89<br>5.1 ± 2.0<br>4.2 ± 2.9	0<br>0.22 ± 0.048<br>0.024 ± 0.011	0 ± 0.001<br>0.24 ± 0.163<br>0.080 ± 0.055	4.5 ± 3.3<br>3.8 ± 2.8<br>3.0 ± 2.1	0.002 ± 0.011<br>0<br>0.001 ± 0.002	0.23 ± 0.121<br>0.36 ± 0.085<br>0.070 ± 0.026	0.082 ± 0.48<br>0<br>0.025 ± 0.159	1.96 ± 0.69<br>3.2 ± 0.71<br>2.3 ± 1.23	0.010 ± 0.004<br>0<br>0
**Jun**	**HAR:** 1.94 ± 4.0<br>**OPD:** 1.85 ± 7.8<br>**RMV:** 1.08 ± 1.47	0.43 ± 0.66<br>0<br>1.52 ± 1.23	1.29 ± 1.03<br>3.3 ± 0.99<br>2.6 ± 1.73	0<br>0.22 ± 0.046<br>0.020 ± 0.013	0 ± 0.001<br>0.25 ± 0.162<br>0.091 ± 0.058	4.2 ± 2.9<br>3.7 ± 2.4<br>3.0 ± 2.0	0.001 ± 0.007<br>0<br>0 ± 0.001	0.21 ± 0.114<br>0.37 ± 0.089<br>0.067 ± 0.025	0.103 ± 0.42<br>0<br>0.022 ± 0.159	1.83 ± 0.65<br>2.9 ± 0.68<br>2.3 ± 1.15	0.010 ± 0.003<br>0<br>0
**Jul**	**HAR:** 0.26 ± 1.41<br>**OPD:** 0.52 ± 4.4<br>**RMV:** 0.84 ± 1.22	0.44 ± 0.60<br>0<br>0.36 ± 0.59	1.63 ± 1.25<br>2.6 ± 0.89<br>2.2 ± 1.06	0<br>0.174 ± 0.043<br>0.006 ± 0.009	0 ± 0.001<br>0.21 ± 0.139<br>0.119 ± 0.078	2.4 ± 1.66<br>4.8 ± 3.1<br>3.7 ± 2.4	0.002 ± 0.010<br>0<br>0.002 ± 0.002	0.181 ± 0.106<br>0.37 ± 0.111<br>0.103 ± 0.025	0.197 ± 0.76<br>0<br>0.057 ± 0.143	1.62 ± 0.63<br>2.9 ± 0.83<br>4.1 ± 1.26	0.010 ± 0.004<br>0<br>0
**Aug**	**HAR:** 0.25 ± 2.0<br>**OPD:** 0.24 ± 1.19<br>**RMV:** 1.16 ± 2.4	0.34 ± 0.50<br>0<br>0.75 ± 1.04	1.61 ± 1.22<br>2.6 ± 0.84<br>4.1 ± 2.2	0<br>0.186 ± 0.050<br>0.011 ± 0.012	0 ± 0.001<br>0.23 ± 0.148<br>0.118 ± 0.076	2.4 ± 1.96<br>4.0 ± 2.7<br>3.8 ± 2.4	0.003 ± 0.013<br>0<br>0.002 ± 0.003	0.178 ± 0.108<br>0.37 ± 0.113<br>0.094 ± 0.031	0.186 ± 0.82<br>0<br>0.068 ± 0.29	1.60 ± 0.64<br>2.4 ± 0.72<br>3.7 ± 1.50	0.0010 ± 0.004<br>0<br>0
**Sep**	**HAR:** 1.69 ± 4.2<br>**OPD:** 1.15 ± 3.8<br>**RMV:** 0.95 ± 1.60	0.44 ± 0.79<br>0<br>0.51 ± 0.87	1.45 ± 1.14<br>4.9 ± 2.3<br>6.9 ± 3.5	0<br>0.20 ± 0.052<br>0.007 ± 0.011	0 ± 0.001<br>0.25 ± 0.155<br>0.123 ± 0.078	3.8 ± 2.7<br>4.6 ± 2.9<br>4.0 ± 2.6	0.003 ± 0.013<br>0<br>0.001 ± 0.002	0.21 ± 0.107<br>0.37 ± 0.108<br>0.084 ± 0.028	0.130 ± 0.61<br>0<br>0.039 ± 0.189	1.73 ± 0.68<br>2.8 ± 0.73<br>3.3 ± 1.32	0.010 ± 0.004<br>0<br>0
**Oct**	**HAR:** 0.71 ± 2.9<br>**OPD:** 0.62 ± 3.8<br>**RMV:** 1.47 ± 2.2	0.37 ± 1.57<br>0<br>0.36 ± 0.52	2.8 ± 2.7<br>4.4 ± 1.64<br>11.5 ± 5.6	0<br>0.26 ± 0.071<br>0.008 ± 0.010	0 ± 0.001<br>0.20 ± 0.130<br>0.131 ± 0.083	2.9 ± 2.5<br>5.1 ± 3.5<br>4.3 ± 2.8	0.004 ± 0.018<br>0<br>0.002 ± 0.003	0.165 ± 0.099<br>0.35 ± 0.105<br>0.096 ± 0.027	0.12 ± 0.59<br>0<br>0.093 ± 0.43	1.59 ± 0.69<br>3.1 ± 1.23<br>3.9 ± 1.23	0.009 ± 0.004<br>0<br>0
**Nov**	**HAR:** 0.065 ± 0.65<br>**OPD:** 0.72 ± 5.5<br>**RMV:** 1.65 ± 2.8	0.22 ± 0.32<br>0<br>0.44 ± 0.77	8.7 ± 5.4<br>7.9 ± 3.5<br>15.6 ± 7.6	0<br>0.24 ± 0.055<br>0.010 ± 0.014	0 ± 0.001<br>0.176 ± 0.126<br>0.121 ± 0.077	1.88 ± 1.71<br>5.1 ± 3.9<br>4.0 ± 2.9	0 ± 0.002<br>0<br>0.002 ± 0.003	0.137 ± 0.076<br>0.36 ± 0.096<br>0.107 ± 0.025	0.039 ± 0.22<br>0<br>0.089 ± 0.33	1.40 ± 0.57<br>3.0 ± 1.07<br>4.3 ± 1.27	0.008 ± 0.003<br>0<br>0
**Dec**	**HAR:** 0.15 ± 1.26<br>**OPD:** 1.58 ± 19.8<br>**RMV:** 1.45 ± 2.6	0.24 ± 0.45<br>0<br>0.39 ± 0.77	9.5 ± 5.8<br>11.1 ± 4.8<br>18.4 ± 9.0	0<br>0.23 ± 0.056<br>0.007 ± 0.011	0 ± 0.001<br>0.154 ± 0.105<br>0.108 ± 0.069	1.77 ± 1.75<br>4.6 ± 3.5<br>3.7 ± 2.5	0 ± 0.002<br>0<br>0.002 ± 0.002	0.131 ± 0.075<br>0.34 ± 0.089<br>0.104 ± 0.025	0.091 ± 0.48<br>0<br>0.089 ± 0.52	1.38 ± 0.63<br>2.7 ± 1.21<br>4.3 ± 1.18	0.008 ± 0.003<br>0<br>0
**Jan**	**HAR:** 1.86 ± 4.9<br>**OPD:** 1.53 ± 8.1<br>**RMV:** 1.48 ± 1.74	0.42 ± 1.22<br>0<br>1.05 ± 1.36	10.2 ± 6.2<br>13.7 ± 6.2<br>15.0 ± 9.2	0<br>0.26 ± 0.072<br>0.018 ± 0.020	0 ± 0.001<br>0.170 ± 0.120<br>0.096 ± 0.066	3.9 ± 3.1<br>3.8 ± 3.1<br>3.1 ± 2.2	0 ± 0.001<br>0<br>0.001 ± 0.003	0.157 ± 0.082<br>0.34 ± 0.091<br>0.086 ± 0.032	0.085 ± 0.44<br>0<br>0.042 ± 0.17	1.63 ± 0.69<br>2.7 ± 0.99<br>3.3 ± 1.54	0.009 ± 0.003<br>0<br>0
**Feb**	**HAR:** 0.54 ± 2.4<br>**OPD:** 1.07 ± 7.7<br>**RMV:** 1.56 ± 1.84	0.31 ± 0.56<br>0<br>0.37 ± 0.65	9.6 ± 6.6<br>10.6 ± 3.9<br>16.5 ± 8.0	0<br>0.25 ± 0.068<br>0.008 ± 0.012	0.001 ± 0.001<br>0.179 ± 0.131<br>0.115 ± 0.075	2.5 ± 2.4<br>4.6 ± 3.5<br>3.6 ± 2.5	0 ± 0.001<br>0<br>0.002 ± 0.002	0.173 ± 0.109<br>0.36 ± 0.099<br>0.101 ± 0.024	0.089 ± 0.45<br>0<br>0.075 ± 0.17	1.53 ± 0.67<br>2.7 ± 0.91<br>4.2 ± 1.13	0.009 ± 0.004<br>0<br>0

Table S4. Means ± standard deviations of calculated CO fluxes (μmol m^{-2} s^{-1}) per month over one year per emission sector at each site. See Table S1 for information on GNFR emission sectors and their abbreviations.

	A	B	C	D	E	F	G	I	J	R
**Mar**	**HAR:** 0	0	0.011 ± 0.007	0	0	0.009 ± 0.006	0	0.002 ± 0.002	0.001	0.001
	**OPD:** 0 ± 0.001	0	0.011 ± 0.008	0	0.002 ± 0.001	0.021 ± 0.015	0	0.025 ± 0.006	0	0
	**RMV:** 0.001 ± 0.001	0.012 ± 0.019	0.033 ± 0.018	0.001 ± 0.001	0.001 ± 0.001	0.012 ± 0.008	0	0.073 ± 0.025	0	0
**Apr**	**HAR:** 0 ± 0.001	0	0.009 ± 0.006	0	0	0.011 ± 0.008	0 ± 0.001	0.003 ± 0.002	0.001	0.001
	**OPD:** 0	0	0.010 ± 0.006	0	0.002 ± 0.002	0.018 ± 0.014	0	0.027 ± 0.007	0	0
	**RMV:** 0.001 ± 0.001	0.022 ± 0.021	0.019 ± 0.013	0.001 ± 0.001	0.001	0.011 ± 0.007	0	0.056 ± 0.029	0	0
**May**	**HAR:** 0 ± 0.001	0	0.002 ± 0.002	0	0	0.014 ± 0.009	0 ± 0.001	0.003 ± 0.002	0.001	0.001
	**OPD:** 0	0	0.005 ± 0.003	0	0.003 ± 0.002	0.019 ± 0.014	0	0.028 ± 0.006	0	0
	**RMV:** 0.002 ± 0.001	0.030 ± 0.020	0.010 ± 0.007	0.001	0.001	0.010 ± 0.007	0	0.046 ± 0.023	0	0
**Jun**	**HAR:** 0	0	0.002 ± 0.001	0	0	0.013 ± 0.008	0	0.003 ± 0.002	0.001	0.001
	**OPD:** 0	0	0.003 ± 0.002	0	0.003 ± 0.002	0.019 ± 0.012	0	0.029 ± 0.006	0	0
	**RMV:** 0.001 ± 0.001	0.025 ± 0.020	0.006 ± 0.004	0.001 ± 0.001	0.001	0.010 ± 0.007	0	0.047 ± 0.021	0	0
**Jul**	**HAR:** 0	0	0.002 ± 0.001	0	0	0.009 ± 0.005	0 ± 0.001	0.003 ± 0.002	0.001	0.001
	**OPD:** 0	0	0.003 ± 0.002	0	0.002 ± 0.001	0.025 ± 0.016	0	0.026 ± 0.007	0	0
	**RMV:** 0.001 ± 0.001	0.006 ± 0.010	0.005 ± 0.003	0	0.001	0.012 ± 0.008	0	0.080 ± 0.024	0	0
**Aug**	**HAR:** 0	0	0.002 ± 0.001	0	0	0.009 ± 0.005	0 ± 0.001	0.002 ± 0.002	0.001	0.001
	**OPD:** 0	0	0.003 ± 0.002	0	0.002 ± 0.002	0.021 ± 0.014	0	0.026 ± 0.008	0	0
	**RMV:** 0.001 ± 0.002	0.012 ± 0.017	0.010 ± 0.006	0 ± 0.001	0.001	0.012 ± 0.008	0	0.072 ± 0.028	0	0
**Sep**	**HAR:** 0	0	0.002 ± 0.001	0	0	0.013 ± 0.007	0 ± 0.001	0.003 ± 0.002	0.001	0.001
	**OPD:** 0	0	0.006 ± 0.005	0	0.003 ± 0.002	0.021 ± 0.013	0	0.028 ± 0.007	0	0
	**RMV:** 0.001 ± 0.001	0.008 ± 0.014	0.017 ± 0.009	0	0.001	0.013 ± 0.009	0	0.065 ± 0.025	0	0
**Oct**	**HAR:** 0	0	0.004 ± 0.004	0	0	0.010 ± 0.007	0 ± 0.001	0.002 ± 0.002	0.001	0.001
	**OPD:** 0	0	0.005 ± 0.004	0	0.002 ± 0.001	0.024 ± 0.016	0	0.025 ± 0.007	0	0
	**RMV:** 0.001 ± 0.002	0.006 ± 0.008	0.028 ± 0.014	0	0.001 ± 0.001	0.014 ± 0.009	0	0.077 ± 0.023	0	0
**Nov**	**HAR:** 0	0	0.012 ± 0.007	0	0	0.008 ± 0.005	0	0.001 ± 0.001	0.001	0.001
	**OPD:** 0	0	0.009 ± 0.007	0	0.002 ± 0.001	0.022 ± 0.016	0	0.024 ± 0.006	0	0
	**RMV:** 0.001 ± 0.002	0.007 ± 0.012	0.038 ± 0.018	0 ± 0.001	0.001	0.013 ± 0.009	0	0.084 ± 0.024	0	0
**Dec**	**HAR:** 0	0 ± 0.001	0.013 ± 0.006	0	0	0.007 ± 0.005	0	0.002 ± 0.001	0.001	0.001
	**OPD:** 0 ± 0.001	0	0.013 ± 0.011	0	0.002 ± 0.001	0.019 ± 0.014	0	0.023 ± 0.006	0	0
	**RMV:** 0.001 ± 0.002	0.006 ± 0.013	0.045 ± 0.022	0	0.001	0.012 ± 0.008	0	0.083 ± 0.022	0	0
**Jan**	**HAR:** 0	0	0.014 ± 0.007	0	0	0.009 ± 0.007	0	0.002 ± 0.001	0.001	0.001
	**OPD:** 0	0	0.016 ± 0.010	0	0.002 ± 0.001	0.016 ± 0.012	0	0.024 ± 0.006	0	0
	**RMV:** 0.001 ± 0.001	0.017 ± 0.022	0.037 ± 0.022	0.001 ± 0.001	0.001	0.010 ± 0.007	0	0.065 ± 0.029	0	0
**Feb**	**HAR:** 0	0 ± 0.001	0.013 ± 0.007	0	0	0.009 ± 0.006	0	0.002 ± 0.002	0.001	0.001
	**OPD:** 0	0	0.012 ± 0.008	0	0.002 ± 0.001	0.019 ± 0.014	0	0.025 ± 0.007	0	0
	**RMV:** 0.001 ± 0.001	0.006 ± 0.011	0.040 ± 0.020	0 ± 0.001	0.001	0.012 ± 0.008	0	0.081 ± 0.021	0	0

Table S5. Means ± standard deviations of calculated CH_4 fluxes (μmol m^{-2} s^{-1}) per month over one year per emission sector at each site. See Table S1 for information on GNFR emission sectors and their abbreviations.

	A	C	D	F	J	K	O
**Mar**	**HAR:** 0 ± 0.001<br>**OPD:** 0<br>**RMV:** 0	0.002 ± 0.002<br>0.002 ± 0.001<br>0.005 ± 0.003	0<br>0.004 ± 0.001<br>0.014 ± 0.003	0<br>0.001<br>0	0 ± 0.001<br>0.009 ± 0.003<br>0.018 ± 0.025	0.001 ± 0.001<br>0<br>0	0.001<br>0<br>0
**Apr**	**HAR:** 0 ± 0.001<br>**OPD:** 0<br>**RMV:** 0	0.002 ± 0.001<br>0.002 ± 0.001<br>0.003 ± 0.002	0<br>0.003 ± 0.001<br>0.008 ± 0.002	0<br>0.001<br>0	0.001 ± 0.001<br>0.010 ± 0.003<br>0.035 ± 0.028	0.001 ± 0.001<br>0<br>0	0.001<br>0<br>0
**May**	**HAR:** 0.001 ± 0.001<br>**OPD:** 0<br>**RMV:** 0	0 ± 0.001<br>0.001<br>0.002 ± 0.001	0<br>0.004 ± 0.001<br>0.007 ± 0.002	0.001<br>0.001 ± 0.001<br>0	0.001 ± 0.001<br>0.010 ± 0.003<br>0.049 ± 0.023	0.001 ± 0.001<br>0<br>0	0.001<br>0<br>0
**Jun**	**HAR:** 0 ± 0.001<br>**OPD:** 0<br>**RMV:** 0	0<br>0.001<br>0.001 ± 0.001	0<br>0.004 ± 0.001<br>0.007 ± 0.002	0.001<br>0.001<br>0	0.001 ± 0.001<br>0.010 ± 0.004<br>0.041 ± 0.027	0.001± 0.001<br>0<br>0	0.001<br>0<br>0
**Jul**	**HAR:** 0<br>**OPD:** 0<br>**RMV:** 0	0<br>0.001<br>0.001	0<br>0.003 ± 0.001<br>0.010 ± 0.002	0<br>0.001 ± 0.001<br>0	0.001 ± 0.001<br>0.008 ± 0.003<br>0.014 ± 0.019	0.001 ± 0.001<br>0<br>0	0.001<br>0<br>0
**Aug**	**HAR:** 0<br>**OPD:** 0<br>**RMV:** 0	0<br>0.001<br>0.002 ± 0.001	0<br>0.003 ± 0.001<br>0.009 ± 0.002	0<br>0.001 ± 0.001<br>0	0.001 ± 0.001<br>0.009 ± 0.003<br>0.023 ± 0.025	0.001 ± 0.001<br>0<br>0	0.001<br>0<br>0
**Sep**	**HAR:** 0 ± 0.001<br>**OPD:** 0<br>**RMV:** 0	0<br>0.001 ± 0.001<br>0.003 ± 0.001	0<br>0.003 ± 0.001<br>0.009 ± 0.002	0<br>0.001<br>0	0.001 ± 0.002<br>0.010 ± 0.003<br>0.016 ± 0.023	0.001 ± 0.001<br>0<br>0	0.001<br>0<br>0
**Oct**	**HAR:** 0 ± 0.001<br>**OPD:** 0<br>**RMV:** 0	0.001 ± 0.001<br>0.001 ± 0.001<br>0.004 ± 0.002	0<br>0.004 ± 0.001<br>0.014 ± 0.003	0<br>0.001 ± 0.001<br>0	0.001 ± 0.001<br>0.009 ± 0.003<br>0.011 ± 0.015	0.001 ± 0.001<br>0<br>0	0.001<br>0<br>0
**Nov**	**HAR:** 0<br>**OPD:** 0<br>**RMV:** 0	0.003 ± 0.002<br>0.002 ± 0.001<br>0.006 ± 0.003	0<br>0.004 ± 0.001<br>0.015 ± 0.003	0<br>0.001<br>0	0 ± 0.001<br>0.009 ± 0.002<br>0.014 ± 0.020	0.001 ± 0.001<br>0<br>0	0.001<br>0<br>0
**Dec**	**HAR:** 0<br>**OPD:** 0<br>**RMV:** 0	0.003 ± 0.002<br>0.002 ± 0.002<br>0.007 ± 0.003	0<br>0.004 ± 0.001<br>0.015 ± 0.003	0<br>0.001<br>0	0.001 ± 0.001<br>0.009 ± 0.003<br>0.011 ± 0.016	0.001 ± 0.001<br>0<br>0	0.001<br>0<br>0
**Jan**	**HAR:** 0 ± 0.001<br>**OPD:** 0<br>**RMV:** 0	0.003 ± 0.002<br>0.003 ± 0.002<br>0.006 ± 0.003	0<br>0.004 ± 0.001<br>0.013 ± 0.004	0<br>0<br>0	0.001 ± 0.002<br>0.009 ± 0.003<br>0.025 ± 0.027	0.001 ± 0.001<br>0<br>0	0.001<br>0<br>0
**Feb**	**HAR:** 0<br>**OPD:** 0<br>**RMV:** 0	0.003 ± 0.002<br>0.002 ± 0.001<br>0.006 ± 0.003	0<br>0.004 ± 0.001<br>0.015 ± 0.003	0<br>0.001<br>0	0.001 ± 0.002<br>0.009 ± 0.003<br>0.011 ± 0.016	0.001 ± 0.001<br>0<br>0	0.001<br>0<br>0

Diurnal Patterns of Modelled Anthropogenic Fluxes

Figure 4 in the main text explores the diurnal patterns of the two dominant contributors to the flux signal for all three species across cities. Figures S16-18 dive deeper into other emission sectors' patterns for CO_2, CO, and CH_4, respectively. Figure S16 shows that smaller but non-negligible contributions from public power and human respiration each have unique diurnal profiles. While public power (Figure S16a) at HAR has a lot of spikes during the day at the same order of magnitude in both summer and winter months, the spikes occur at different times. There are more spikes, which also reach a higher magnitude, in winter at OPD (Figure S16b); the summer profile at OPD shows a peak around 05:00 UTC and 18:00 UTC. There is a clear increase in public power fluxes throughout the day in winter at RMV with the largest peak occurring at 22:00 UTC while the RMV summer profile remains steady throughout the day. Because human respiration (Figure S16d-f) emissions have no applied temporal profile, it can be assumed that any patterns observed are due to footprint location, extent, and weighting of specific areas.

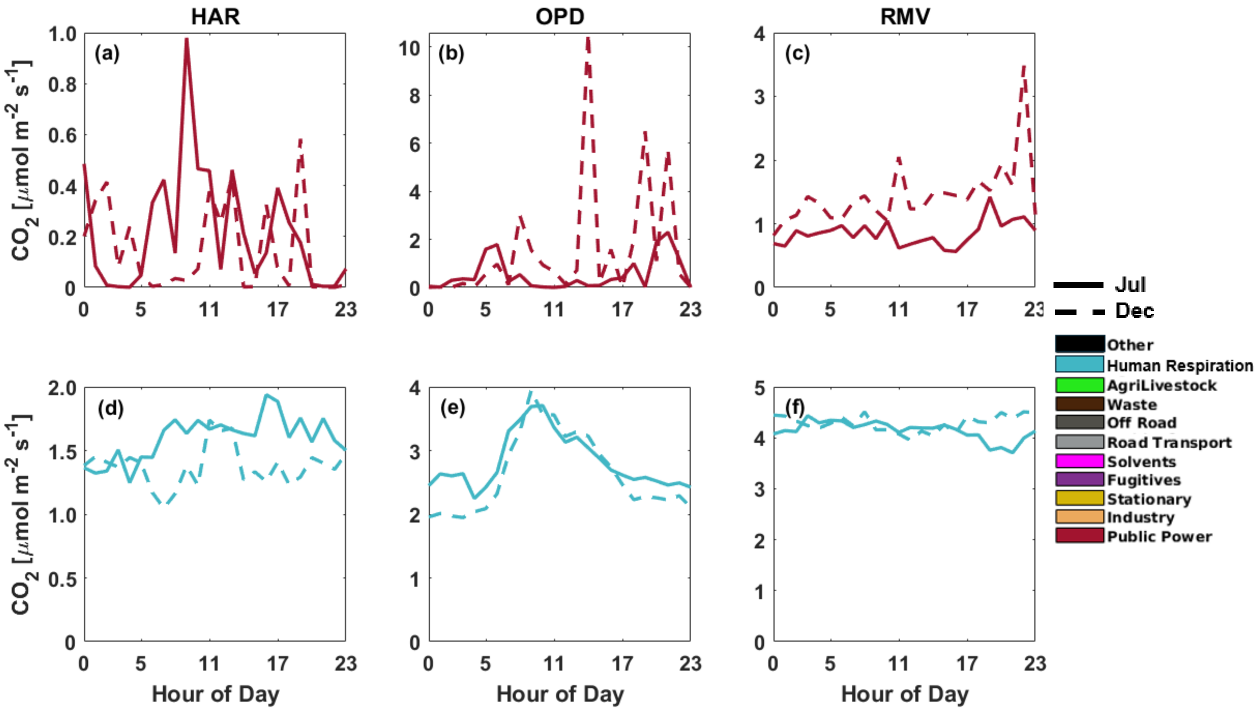

Figure S16. Diurnal trends (UTC) in Jul 2023 (solid) and Dec 2023 (dashed) for two additional important contributors to CO_2 fluxes from public power (top row) and human respiration (bottom row) at (a)/(d) HAR, (b)/(e) OPD, and (c)/(f) RMV.

Figure S17a indicates that offroad transport CO emissions at HAR follow a similar diurnal profile in summer and winter with the winter profile lagging behind by no more than an hour; both show a sharp increase in the early morning hours and remain elevated during the day, only decreasing in the evening hours. Industrial combustion is nonzero throughout the day but does not increase very much in either season. Figure S17b shows that stationary combustion CO emissions at OPD have a different pattern from CO_2 fluxes (Figure 4b) despite having the same temporal profile. Figure S17b also shows that emissions from solvents increase from zero from 04:00-06:00 UTC in summer (05:00-07:00 UTC in winter) and remain elevated until 17:00 UTC. Figure S17c demonstrates that road transport CO fluxes at RMV are nonzero and behave

similarly to road transport CO_2 emissions as shown in Figure 4c in the main text. Emissions from industrial combustion are higher in winter than in summer during the day while the inverse is true at night.

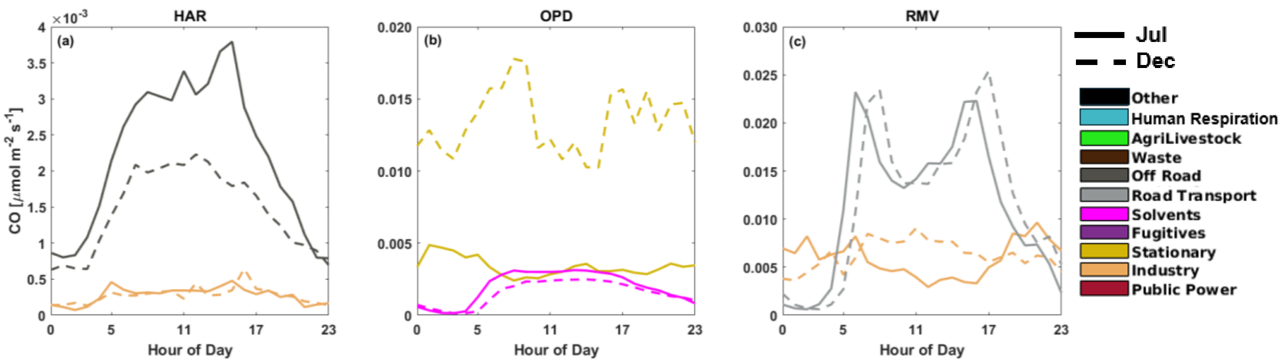

Figure S17. Diurnal trends (UTC) in Jul 2023 (solid) and Dec 2023 (dashed) for two additional important contributors to CO fluxes at (a) HAR, (b) OPD, and (c) RMV.

Figure S18 indicates that although contributions to methane emissions from remaining sectors are low, they can still exhibit interesting diurnal behavior. Figure S18a shows that waste emissions at HAR have small peaks at different hours of the day in each season, and both seasons show lower values during the day than at night. Methane fluxes from human respiration are low in magnitude but similar to CO_2 fluxes in Figure S16d, as expected. Figure S18b indicates that road transport CH_4 fluxes are lower in winter than in summer at OPD and behave similarly to the other two species as observed in Figures 4b and 4e. Figure S18c shows that fugitive fluxes are slightly higher in winter than summer and constantly slightly elevated all day in both seasons.

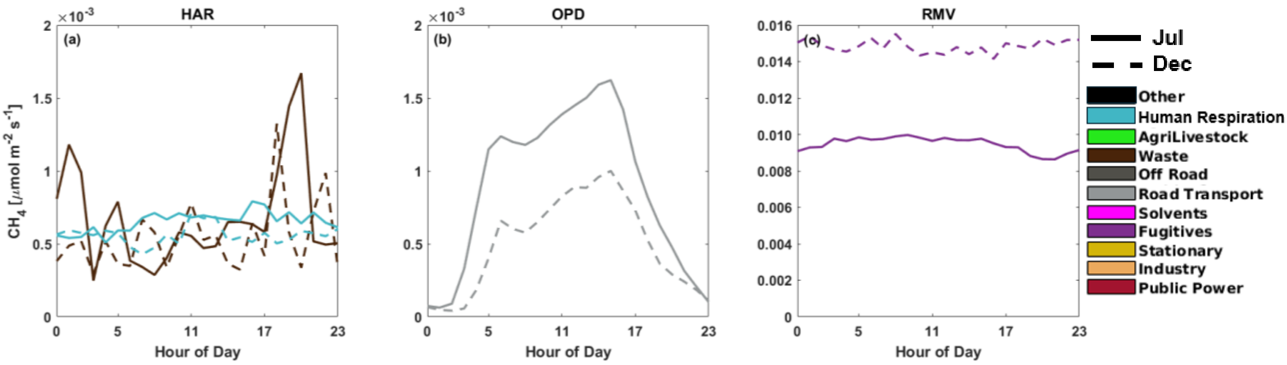

Figure S18. Diurnal trends (UTC) in Jul 2023 (solid) and Dec 2023 (dashed) for two additional important contributors to CH_4 fluxes at (a) HAR, (b) OPD, and (c) RMV.

Other Emissions

The 'other' category in the Zurich emission inventory accounts for sources such as smoking, animals, fireworks and accidental fires, and thunderstorms. The contribution of 'other' sources to the flux signal of any species at HAR is extremely low but not zero, with the exception of contribution to CH_4 (Figure S19b).

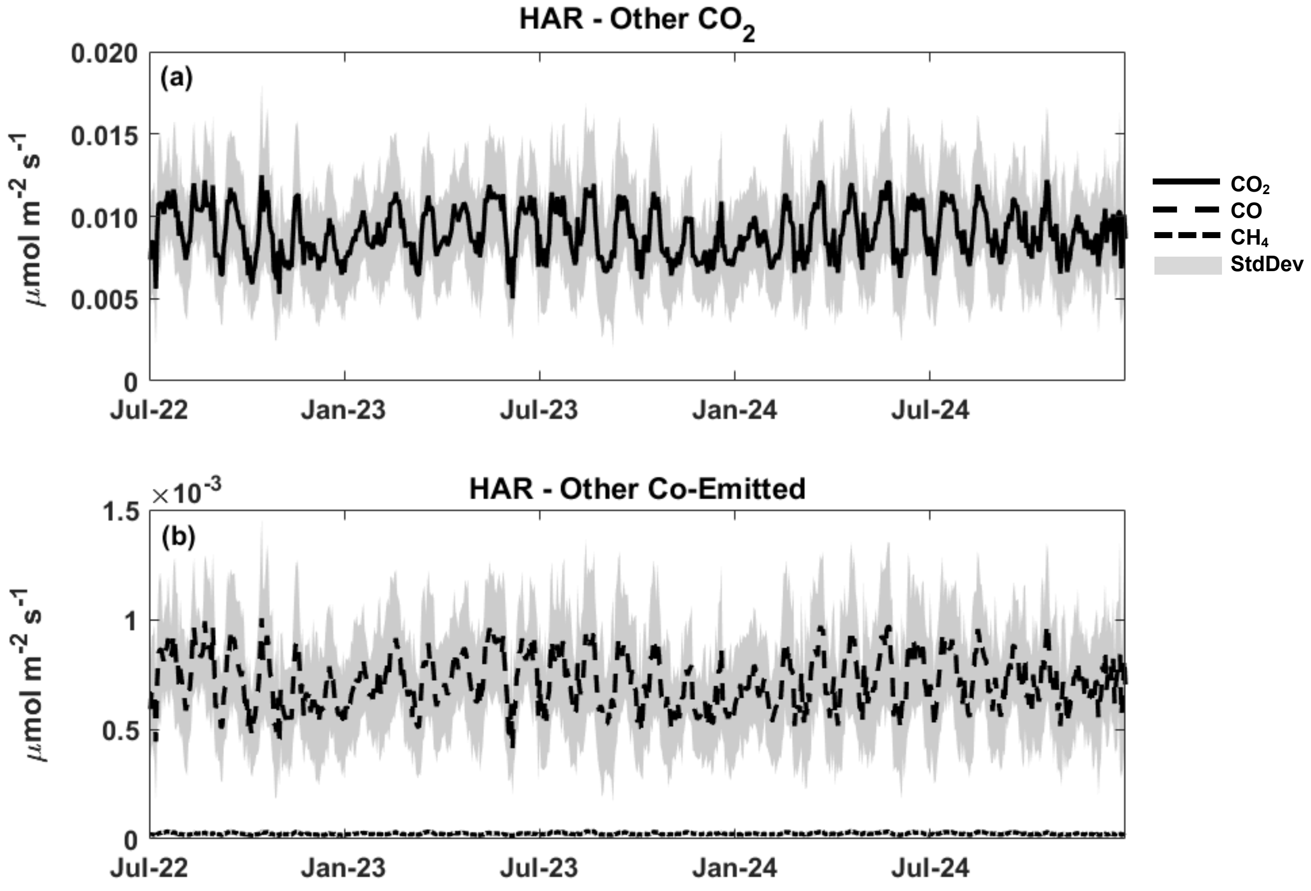

Figure S19. Mean (lines) and standard deviations (shaded area) of monthly diurnal modelled 'other' fluxes at HAR of (a) CO_2 and (b) CO and CH_4.

References

1. Lindberg, F.; Grimmond, C. S. B.; Gabey, A.; Huang, B.; Kent, C. W.; Sun, T.; Theeuwes, N. E.; Järvi, L.; Ward, H. C.; Capel-Timms, I.; Chang, Y.; Jonsson, P.; Krave, N.; Liu, D.; Meyer, D.; Olofson, K. F.; Tan, J.; Wästberg, D.; Xue, L.; Zhang, Z. Urban Multi-Scale Environmental Predictor (UMEP): An Integrated Tool for City-Based Climate Services. *Environmental Modelling & Software* **2018**, *99*, 70–87. DOI:10.1016/j.envsoft.2017.09.020.

2. Macdonald, R. W.; Griffiths, R. F.; Hall, D. J. An Improved Method for the Estimation of Surface Roughness of Obstacle Arrays. *Atmospheric Environment* **1998**, *32* (11), 1857–1864. DOI:10.1016/s1352-2310(97)00403-2.

3. Kljun, N.; Calanca, P.; Rotach, M. W.; Schmid, H. P. A Simple Two-Dimensional Parameterisation for Flux Footprint Prediction (FFP). *Geoscientific Model Development* **2015**, *8* (11), 3695–3713. DOI:10.5194/gmd-8-3695-2015.

4. Kuenen, J.; Dellaert, S.; Visschedijk, A.; Jalkanen, J.-P.; Super, I.; Denier van der Gon, H. CAMS-Reg-V4: A State-of-the-Art High-Resolution European Emission Inventory for Air Quality Modelling. *Earth System Science Data* **2022**, *14* (2), 491–515. DOI:10.5194/essd-14-491-2022.

5. Super, I.; Dellaert, S. N.; Visschedijk, A. J.; Denier van der Gon, H. A. Uncertainty Analysis of a European High-Resolution Emission Inventory of CO2 and CO to Support Inverse Modelling and Network Design. *Atmospheric Chemistry and Physics* **2020**, *20* (3), 1795–1816. DOI:10.5194/acp-20-1795-2020.

6. Super, I.; Denier van der Gon, H. A.; Dröge, R. *Downscaling tool to construct city emission inventories.* R12516; Netherlands Organisation for Applied Scientific Research, Department of Climate, Air and Sustainability: Utrecht, Netherlands, 2025. https://publications.tno.nl/publication/34645367/HuOA6az5/TNO-2025-R12516.pdf

7. European Union's Copernicus Land Monitoring Service. CLCplus Backbone 2021 (raster 10 m), Europe, 3-yearly, Sept. 2024, 2024. https://doi.org/10.2909/71fc9d1b-479f-4da1-aa66-662a2fff2cf7.

8. European Union's Copernicus Land Monitoring Service. Impervious Built-up 2018 (raster 10 m and 100 m), Europe, 3-yearly, Sep. 2024, 2020. https://doi.org/10.2909/3e412def-a4e6-4413-98bb-42b571afd15e.

9. European Union's Copernicus Land Monitoring Service. CORINE Land Cover 2018 (vector/raster 100 m), Europe, 6-yearly, Sep. 2020, 2020. https://doi.org/10.2909/960998c1-1870-4e82-8051-6485205ebbac.

10. Jedlicka, K.; Hajek, P.; Cada, V.; Martolos, J.; Stastny, J.; Beran, D.; Kolovsky, F.; Kozhukh, D. Open Transport Map — Routable OpenStreetMap. *2016 IST-Africa Week Conference* **2016**, 1–11. DOI:10.1109/istafrica.2016.7530657.

11. Pesaresi, M.; Politis, P. *GHS-BUILT-V R2023A - GHS built-up volume grids derived from joint assessment of Sentinel2, Landsat, and global DEM data, multitemporal (1975-2030)* (Dataset), European Commission, Joint Research Centre (JRC), April 13, 2023. DOI:10.2905/AB2F107A-03CD-47A3-85E5-139D8EC63283

12. Schiavina, M.; Freire, S.; Carioli, A.; MacManus, K. *GHS-POP R2023A - GHS population grid multitemporal (1975-2030)*, European Commission, Joint Research Centre (JRC), April 13, 2023. DOI:10.2905/2FF68A52-5B5B-4A22-8F40-C41DA8332CFE
